# Acceptability of Carraguard Vaginal Microbicide Gel among HIV-Infected Women in Chiang Rai, Thailand

**DOI:** 10.1371/journal.pone.0014831

**Published:** 2011-09-07

**Authors:** Sara J. Whitehead, Catherine McLean, Supaporn Chaikummao, Sarah Braunstein, Wat Utaivoravit, Janneke H. van de Wijgert, Philip A. Mock, Taweesap Siraprapasiri, Barbara A. Friedland, Peter H. Kilmarx, Lauri E. Markowitz

**Affiliations:** 1 Thailand Ministry of Public Health–U.S. Centers for Disease Control and Prevention Collaboration, Nonthaburi, Thailand; 2 National Center for HIV/AIDS, Viral Hepatitis, STD, and TB Prevention, U.S. Centers for Disease Control and Prevention, Atlanta, Georgia, United States of America; 3 Population Council, New York, New York, United States of America; 4 Chiang Rai Hospital, Chiang Rai, Thailand; 5 Academic Medical Center of the University of Amsterdam and Amsterdam Institute for Global Health and Development, Amsterdam, The Netherlands; Tulane University, United States of America

## Abstract

**Background:**

Few studies of microbicide acceptability among HIV-infected women have been done. We assessed Carraguard® vaginal gel acceptability among participants in a randomized, controlled, crossover safety trial in HIV-infected women in Thailand.

**Methodology/Principal Findings:**

Participants used each of 3 treatments (Carraguard gel, methylcellulose placebo gel, and no product) for 7 days, were randomized to one of six treatment sequences, and were blinded to the type of gel they received in the two gel-use periods. After both gel-use periods, acceptability was assessed by face-to-face interview. Responses were compared to those of women participating in two previous Carraguard safety studies at the same study site. Sixty women enrolled with a median age of 34 years; 25% were sexually active. Self-reported adherence (98%) and overall satisfaction rating of the gels (87% liked “somewhat” or “very much”) were high, and most (77%) considered the volume of gel “just right.” For most characteristics, crossover trial participants evaluated the gels more favorably than women in the other two trials, but there were few differences in the desired characteristics of a hypothetical microbicide. Almost half (48%) of crossover trial participants noticed a difference between Carraguard and placebo gels; 33% preferred Carraguard while 12% preferred placebo (p = 0.01).

**Conclusions/Significance:**

Daily Carraguard vaginal gel use was highly acceptable in this population of HIV-infected women, who assessed the gels more positively than women in two other trials at the site. This may be attributable to higher perceived need for protection among HIV-infected women, as well as to study design differences. This trial was registered in the U.S. National Institutes of Health clinical trials registry under registration number NCT00213044.

## Introduction

The development of a safe, effective topical microbicide for HIV prevention is a major public health priority [1]. Over 40 compounds are being evaluated as topical vaginal microbicides, including 12 products currently in clinical trials [2]. Microbicides are intended for use by women at risk of sexually transmitted HIV, but once marketed they may also be used by HIV-infected women either because they are unaware of their HIV status, to protect themselves against other sexually transmitted infections (STI), or to prevent HIV transmission to a sex partner.

The eventual uptake and consistent use of microbicides will depend on their acceptability among both women and men, and acceptability data are critical to inform both product formulation and social marketing messages [3]. Despite a rapidly growing literature on microbicide acceptability, there have been few studies among HIV-infected women [3]-[9]. We report acceptability results from a safety trial with crossover design of Carraguard® vaginal gel among HIV-infected women in Chiang Rai, Thailand, and present a comparison with acceptability data from two previous Carraguard trials at the same site.

A recently completed phase III trial of Carraguard did not demonstrate efficacy in preventing HIV acquisition [10], but combination products including Carraguard and anti-retroviral drugs are currently under development at the Population Council. Acceptability findings regarding Carraguard may therefore be useful both for these next-generation candidate microbicides and for other gels with similar properties.

## Methods

### Ethics Statement

This study was conducted according to the principles expressed in the Declaration of Helsinki, and was approved by the Institutional Review Boards of the Population Council, Centers for Disease Control and Prevention, and the Ethical Review Committee of the Thailand Ministry of Public Health. Written informed consent was obtained from all study participants. The protocol for this trial is available as supporting information; see Protocol S1.

### Study design

Acceptability data were collected as part of a 3-month, randomized, placebo-controlled crossover trial conducted to evaluate the safety and acceptability of Carraguard among HIV-infected women (hereafter referred to as “crossover trial”). Each woman participated in each of 3 study interventions (Carraguard, methylcellulose placebo, and no product) over three consecutive months with the order of interventions randomly assigned. When using study gels, women were blinded to the type of gel they received. Carraguard and placebo are both clear and odorless gels packaged in a single-use Microlax®-type applicator (Norden Pac International AB, Kalmar, Sweden). This applicator was shown in previous studies to dispense about 5 ml of gel [11]. Participants attended the clinic at day 0, 7, and 14 of each study month for interview, examination and specimen collection. They were asked to apply study gel vaginally once daily for 7 days starting after menses were completed (day 0) during the two gel months. Full methods and safety findings are reported in detail elsewhere [12].

### Study population

Women were recruited from HIV care clinics and support groups in five districts of Chiang Rai province in northern Thailand. Eligibility criteria included aged 18-50 years; CD4+ cell count 51–500/mm^3^; not currently on antiretroviral therapy (ART); and either being sexually abstinent or having a single male partner who was confirmed HIV-infected and aged 18 years or older. At study initiation, eligibility for ART in public clinics was limited; women were referred for ART at the time of study screening and encouraged to initiate ART as soon as available, regardless of study participation. During the study, eligibility expanded substantially and all eligible women accessed ART during or shortly after the study.

### Acceptability data collection

After completing both gel-use periods, women completed an interviewer-administered questionnaire that included both structured and open-ended questions about the participants' views on product attributes and use of the gels. Most of these questions addressed both gels simultaneously, as previous studies had shown no difference in acceptability responses between Carraguard and placebo groups [13], [14]. However, the previous studies were traditional parallel arm studies in which participants were randomized to either Carraguard or placebo. Since this crossover trial was the first time that individual women used both Carraguard and placebo, additional questions asked women to compare the two gels they had used. At the time these questions were asked, the women did not yet know which of the two gels was Carraguard and which was placebo. Adherence was assessed by participant self-report in face-to-face interviews and by counting returned used and unused applicators (returned in separate bags).

### Data analysis

Data were double-entered into an Access database (Microsoft, Inc., Redmond, Washington, USA). Codes for open-ended questions were initially developed by trial staff and were reviewed and revised by staff members at the Population Council in New York City. Data were analyzed using SAS statistical software package (SAS version 9.1, SAS Institute, Cary, North Carolina, USA). Chi-square tests were used to evaluate differences between groups.

### Comparison with previous Carraguard studies in Thailand

Some acceptability questions in the crossover trial were asked in an identical manner in two previous studies of Carraguard safety and acceptability at the same site in Thailand; for these questions, comparisons are presented here. The first study was conducted to assess safety and acceptability among 165 low-risk, HIV-uninfected women followed for one year (hereafter referred to as “phase II trial”)[14], [15]. Participants were asked to apply Carraguard or placebo once per day in the evening, and if sex took place, within one hour before sex together with condoms; 98% of participants were married and sexually active. The second study enrolled 55 low-risk, HIV-uninfected, sexually active couples for 6 months, primarily to assess safety of exposure during intercourse and acceptability among male partners; safety and acceptability among female participants were also evaluated [13], [16]. Couples were asked to apply Carraguard or placebo within one hour before sex; condoms and counseling were provided but condoms were rarely used in the trial. We present acceptability responses from female participants in the phase II and couples trials concurrently with results of the crossover trial for comparison.

## Results

### Crossover study population

Between 2002 and 2004, 222 women were screened and 60 were enrolled in the trial; one woman was lost to follow up after she completed the acceptability assessment. Enrolled women had a median age of 34 years, had been diagnosed with HIV a median of 44 months previously, and had a median CD4+ lymphocyte count of 296 cells/mL (Table 1). Seventeen (28%) women were married, with the remainder either widowed (60%) or divorced (12%). The 15 (25%) women who were sexually active at the time of the trial reported having had sex a median of 4 times in the previous month, and median condom use in the previous month was 4 times. No participants reported anal or oral sex in the previous month.

**Table 1:** Baseline demographic, behavioral, and clinical characteristics.

Characteristic	Participants (N = 60)
Age in years, median (Q1–Q3)	34 (30–40)
Marital status	
Widowed	36 (60%)
Married	17 (28%)
Separated/Divorces	7 (12%)
Ever accepted money/gifts for sex	8 (13%)
Any vaginal product use in past month	10 (17%)
Vaginal sex in past month^1^	15 (25%)
If sex, number of sex acts last mo, median (Q1–Q3)	4 (2–9)
If sex, times condom used last mo, median (Q1–Q3)	4 (2–6)
Current use of contraception^2^	30 (50%)
Years since first HIV+ test, median (Q1–Q3)	4.6 (2.7–7.1)
CD4 cell count, median (Q1–Q3)	296 (168–349)
Plasma HIV viral load (log_10_ copies/ml), median (Q1–Q3)	4.6 (4.1–5.2)

1 No anal or oral sex reported.

2 Although only 25% of participants were sexually active at the time of the trial, women who had a permanent method of contraception are also reported as current contraceptive users.

### Adherence

Reported adherence to gel use was high, with 59 (98%) women using at least 6 of 7 scheduled doses during each gel use period. Only one woman interrupted gel use for reasons unrelated to adverse effects.

### Acceptability

Overall satisfaction rating of the gels was high, with 22 (37%) and 30 (50%) participants reporting they liked the gel “very much” and “somewhat,” respectively, and 60 (100%) stating they would recommend it to a friend (Table 2). Most participants described the applicator as appealing (72%) or neutral (25%), and found the gel easy to apply (83%). The gel volume was assessed as “just right” by 46 (77%) women and “too much” by 14 (23%), while 55 (92%) women considered the extra lubrication provided by gel an advantage. Only 17 (28%) women believed that it would be possible to use gel without their partner being aware. In response to open-ended questions about how they would change the product if they could, 45 women had no suggested changes, 12 suggested softening the applicator tip, and 10 suggested a smaller gel volume.

**Table 2:** Carraguard and placebo gel acceptability among HIV-positive and negative women in 3 trials in Chiang Rai, Thailand^1^.

	HIV-positive women, crossover trial	HIV-negative women, phase II trial	HIV-negative women, couples trial	
	2003–2004	2000–2001	2001–2002	
Characteristic	N = 60	N = 157	N = 54	p-value
Overall satisfaction rating of gel				<0.0001
Liked very much	22 (37%)	18 (12%)	12 (22%)	
Liked somewhat	30 (50%)	87 (55%)	38 (70%)	
Neutral	4 (7%)	48 (31%)	4 (7%)	
Disliked some/very much	4 (7%)	4 (2%)	0	
Would recommend gel to a friend	60 (100%)	157 (100%)	54 (100%)	–
Volume of gel				<0.0001
Too much	14 (23%)	97 (62%)	29 (55%)	
Just right	46 (77%)	59 (38%)	24 (45%)	
Not enough	0	1 (1%)	0	
Applicator				0.02
Appealing	43 (72%)	77 (49%)	28 (52%)	
Neutral	15 (25%)	73 (46%)	26 (48%)	
Unappealing	2 (3%)	7 (4%)	0	
Ease of gel application				0.002
Somewhat/very easy	50 (83%)	101 (64%)	34 (63%)	
Neutral	10 (17%)	51 (32%)	19 (35%)	
Somewhat/very difficult	0	5 (3%)	1 (2%)	
Extra lubrication an advantage	55 (92%)	117 (74%)	52 (96%)	<0.0001
Covert use possible	17 (28%)	71 (45%)	9 (17%)	0.0003

1 Acceptability responses are presented from the visit after completion of 2 gel use periods for participants in the crossover trial, and from the closing visit for the phase II and couples trials.

Acceptability responses varied among the three Thai safety trial populations for most characteristics (Table 2). For example, there was a significant difference among groups in the overall satisfaction rating of gel; more women in the crossover trial said they “liked the gel very much” compared to women in the other two studies. There were also differences in the proportion of women considering covert use possible, and judging the volume of gel as too much. For most parameters, participants in the crossover trial evaluated the gels more favorably than those in the other two trials.

### Desired characteristics of a microbicide

Crossover trial participants rated the following characteristics as most important in a microbicide: the partner being unable to tell gel is there (77% very important, 15% somewhat important), being personally unable to tell gel is there (73% very important, 13% somewhat important), providing extra lubricant (58% very important, 37% somewhat important), and making the vagina feel tight (57% very important, 30% somewhat important) (Table 3). There were statistically significant differences among the 3 trial populations for only three of the ten microbicide characteristics assessed: extra lubricant, giving a warm feeling, and having a good taste.

**Table 3:** Desired product characteristics reported by HIV-positive and negative women in 3 trials in Chiang Rai, Thailand.

	Crossover trial (HIV-positives)	Phase II trial (HIV-negatives)	Couples trial (HIV-negatives)	
	2003–2004	2000–2001	2001–2002	
Characteristic	N = 60	N = 157	N = 54	p
Extra lubricant				0.0004
Very important	35 (58%)	65 (41%)	32 (59%)	
Somewhat important	22 (37%)	56 (36%)	20 (37%)	
Not important	2 (3%)	36 (23%)	2 (4%)	
Dries vagina				0.12
Very important	1 (2%)	19 (12%)	2 (4%)	
Somewhat important	4 (7%)	8 (5%)	5 (9%)	
Not important	55 (92%)	128 (82%)	46 (85%)	
Gives warm feeling				0.005
Very important	10 (17%)	9 (6%)	5 (9%)	
Somewhat important	19 (32%)	33 (21%)	22 (41%)	
Not important	30 (50%)	114 (73%)	26 (48%)	
Makes vagina feel tight				0.21
Very important	34 (57%)	88 (56%)	23 (43%)	
Somewhat important	18 (30%)	35 (22%)	21 (39%)	
Not important	8 (13%)	32 (20%)	10 (18%)	
Cannot tell gel is there				0.17
Very important	44 (73%)	92 (59%)	31 (57%)	
Somewhat important	8 (13%)	15 (28%)	15 (28%)	
Not important	8 (13%)	19 (12%)	8 (15%)	
Partner cannot tell gel is there				0.25
Very important	46 (77%)	108 (69%)	33 (61%)	
Somewhat important	9 (15%)	39 (25%)	16 (30%)	
Not important	4 (7%)	10 (6%)	5 (9%)	
Slows time to ejaculation				0.13
Very important	11 (18%)	23 (15%)	12 (22%)	
Somewhat important	16 (27%)	29 (18%)	18 (33%)	
Not important	33 (55%)	104 (66%)	24 (44%)	
Speeds time to ejaculation				0.77
Very important	17 (28%)	34 (22%)	10 (18%)	
Somewhat important	16 (27%)	49 (31%)	17 (32%)	
Not important	27 (45%)	74 (47%)	27 (50%)	
Smells good				0.49
Very important	27 (45%)	64 (41%)	17 (32%)	
Somewhat important	17 (28%)	44 (28%)	23 (43%)	
Not important	16 (27%)	48 (31%)	14 (26%)	
Tastes good				0.002
Very important	19 (32%)	15 (10%)	5 (9%)	
Somewhat important	11 (18%)	40 (26%)	10 (18%)	
Not important	29 (48%)	100 (64%)	39 (72%)	

### Comparison of Carraguard and placebo gels by crossover trial participants

Almost half of crossover trial participants (48%) reported that they noticed a difference between the Carraguard and placebo gels (Table 4). Among the 29 women who reported a difference, 15 reported on open-ended questioning that placebo gel leaked more, 6 that placebo felt more viscous, 5 that Carraguard felt more natural, and 4 that Carraguard felt more viscous. A total of 20 (33%) women preferred Carraguard while 7 (12%) preferred placebo (p = 0.01), with no significant difference by order of gel received. Most women who preferred Carraguard over placebo did so because they perceived that it was less messy (12 responses) and felt natural (9 responses) (Table 5). More women on placebo reported leakage or messiness (16 responses) as their least liked feature.

**Table 4:** Comparisons between Carraguard and placebo gels by HIV-positive women in the crossover trial, Chiang Rai, Thailand.

	All participants	Carraguard-placebo sequence^1^	Placebo-Carraguard sequence^2^
Question	N = 60	N = 30	N = 30
Noticed a difference between gels	29 (48%)	17 (57%)	12 (40%)
Gel preferred^3^			
Carraguard	20 (33%)	12 (40%)	8 (27%)
Placebo	7 (12%)	4 (13%)	3 (10%)
No preference/no difference	33 (55%)	14 (47%)	19 (63%)
Most common differences reported^4^			
Placebo leaked more	15 (25%)	5 (17%)	10 (33%)
Placebo more viscous	6 (10%)	3 (10%)	3 (10%)
Carraguard felt more natural	5 (8%)	1 (3%)	4 (13%)
Carraguard more viscous	4 (7%)	0	4 (13%)

1 Includes women randomized to the following sequences: Carraguard-placebo-no gel; Carraguard-no gel-placebo; and no gel-Carraguard-placebo. Women in the gel use groups were blinded to the type of gel they received.

2 Includes women randomized to the following sequences: placebo-Carraguard-no gel; placebo-no gel-Carraguard; and no gel-placebo-Carraguard. Women in the gel use groups were blinded to the type of gel they received.

3 The difference between the number of women preferring Carraguard over placebo was statistically signficant with p = 0.01; there was no significant difference in gel preference by randomization sequence.

4 Responses are from open-ended questions.

**Table 5:** Open-ended comments regarding gel preferences by crossover trial participants^1^.

	Preferred Carraguard (n = 20)	Preferred placebo (n = 7)
**Best liked features**	Less messy (12 responses)	Less messy (3 responses)
	Natural/no sensation (9 responses)	Natural/no sensation (2 responses)
	Lubrication (5 responses)	Lubrication (2 responses)
**Least liked features**	Leakage/messiness (8 responses)	Leakage/messiness (16 responses)
	Too sticky/viscous (1 response)	Too sticky/viscous (5 responses)
		Too much volume (3 responses)
		Too wet/too watery (3 responses)

1 Total responses do not add up to the number of women with preference for one gel (n = 27) because some women gave more than one response to each question.

### Role of microbicides for HIV-infected women

When asked what role microbicides might play for them as HIV-infected women, participants most commonly responded that microbicides could protect them from infection with another strain of HIV or other STI (51 responses), and that it might protect their partner from HIV acquisition (13 responses). On the other hand, when asked, “If microbicides provided the same protection as condoms, do you think men would be less willing to use condoms?” 57 (95%) women responded yes. A majority of women (68%) reported being willing to pay at least the equivalent price as condoms for a microbicide.

## Discussion

Overall acceptability of study gels was high among this population of HIV-infected women. Our findings contribute to the small body of literature evaluating the acceptability of microbicide candidates among HIV-infected women [4], [7], [8], [17].

Acceptability responses of women in this crossover trial were broadly positive, as they were among women in two previous Carraguard safety studies in the same northern Thai community and in a similar trial including HIV-infected women in Durban [7]. Although the questions were not identical between the trials, similar proportions of HIV-infected women participants in Chiang Rai and Durban reported they liked the product (87% in Chiang Rai vs. 93% in Durban) and believed it could be used covertly (28% and 30% respectively). Given the known regional differences in preferences for vaginal lubrication during sex [18], it is not surprising that there were also differences between the Thai and South African participants in the importance assigned to a microbicide that provides extra lubrication (95% in Chiang Rai vs. 69% in Durban) and that dries the vagina (9% vs. 41%).

In comparing responses of women in the crossover trial to those of the other two Carraguard trials at the Chiang Rai site, women in the crossover trial rated the study gels more positively than women in the other studies for most characteristics. This may be partly attributable to their HIV-infected status: a higher perception of risk and a personal experience of the impact of HIV disease may make women more enthusiastic about prevention technologies and more tolerant of inconveniences of product use such as leakage [17]. However, important differences in participant characteristics, trial design, duration, and study requirements are also likely to have contributed to the different responses. In particular, participants in the phase II and couples trials were in long-term, stable partnerships compared to only a quarter of participants in the crossover trial; consistent condom use concurrent with microbicide use was strongly counselled in the phase II study and crossover studies but not in the couples study; and the duration of product use varied from one year in the phase II trial and 6 months in the couples trial to two one-week periods in the crossover trial. There was evidence in both the phase II and the couples studies that acceptability responses changed over time [13], [14]. The observed differences in acceptability responses among these three trials of the same product in the same community and cultural context suggests that differences in study design and context of use are important contributors to womens' responses to a product. This suggests that even if standardized acceptability measures are used, comparisons of the acceptability of a candidate microbicide in different populations, or comparison of different microbicide candidates, must be interpreted with great caution.

A unique feature of the crossover trial was that, by design, each participant used both Carraguard and placebo gel. Carraguard and its methylcellulose placebo were both clear and odorless gels packaged in identical opaque applicators, but due to differences in the rheological properties of the two compounds the placebo had somewhat lower viscosity and may show other differences in rheological properties in the face of shear stress. About half the women in this trial noticed a difference between the two gels, and most of these women preferred Carraguard over placebo. Placebo was more commonly reported to cause leakage, and Carraguard was described as less messy and as feeling more natural. These findings suggest that even relatively subtle differences in the biophysical properties of candidate microbicide gels are perceived by at least some women. There may be an optimal set of such properties with respect to product acceptability, which should be considered alongside the vaginal coating and distribution considerations that currently inform formulation science early in the product development cycle [9], [19], [20].

Our study had a number of limitations. This small, single-site safety trial enrolled a group of highly motivated participants who met multiple inclusion criteria, and thus results may not be representative of HIV-infected women in the broader community. Participants used gel for only two seven-day periods, and applied it once daily in the evening rather than before sex which may more closely simulate eventual recommended use of the product (although antiretroviral-based microbicides may be developed for daily rather than pre-coital use). Participants were strongly counseled to use condoms consistently during the trial, so acceptability responses for sexually active participants reflect concurrent gel and condom use only, rather than gel use alone. In addition, only a quarter of the study population was sexually active during the trial, so many responses related to gel use during sex were hypothetical. Finally, acceptability data were collected in face-to-face interviews which may have led to social desirability biases in women's responses.

Although topical microbicides currently under development are intended for use by HIV-uninfected women, the HIV-infected women participating in this trial identified an interest and need for microbicides, including protection against other STI and protection of partners from HIV infection. With the current focus on and promise of antiretroviral-based microbicides [22], the potential utility of non-antiretroviral microbicides for STI protection in both HIV-infected and uninfected women should remain under consideration and research planned accordingly. On a cautionary note, most trial participants believed an effective microbicide would make men less likely to use condoms; this concerning finding was also reported by Ramjee et al. [7] and suggests that educational and marketing messages will need to be carefully tailored around hierarchies of risk, especially in high-HIV prevalence settings.

It has been recommended that microbicide candidate product safety be demonstrated among HIV-infected women before widespread marketing [21]. Safety trials such as this one therefore offer an ideal opportunity to assess microbicide acceptability prior to introduction, so that potential microbicide uses and marketing messages associated with microbicides among women living with HIV and their partners can be considered.

## Supporting Information

Protocol S1 Trial protocol.
(0.48 MB DOC)

## References

[1] Harrison P, Rosenberg S, Bowcut J (2003). Topical microbicides for disease prevention: Status and challenges.. Clin Infect Dis.

[2] Alliance for Microbicide Development. Microbicide Pipeline Summary Report.. http://www.microbicide.org/uploads/3/1/2/1/3121935/microbicide_pipeline_update_1_oct_2009.pdf.

[3] Mantell JE, Myer L, Carballo-Diéguez A, Stein Z, Ramjee G (2005). Microbicide acceptability research: Current approaches and future directions.. Soc Sci Med.

[4] El-Sadr WM, Mayer KH, Maslankowski L, Hoesley C, Justman J (2006). Safety and acceptability of celulose sulfate as a vaginal microbicide in HIV-infected women.. AIDS.

[5] Mayer KH, Maslankowski LA, Gai F (2006). Safety and tolerability of tenofovir vaginal gel in abstinent and sexually active HIV-infected and uninfected women.. AIDS.

[6] Jespers VA, Van Roey JM, Beets GI, Buvé AM (2007). Dose-ranging phase 1 study of TMC120, a promising vaginal microbicide, in HIV-negative and HIV-positive female volunteers.. J Acquir Immune Defic Syndr.

[7] Ramjee G, Morar NS, Braunsten S, Friedland B, Jones H (2007). Acceptability of Carraguard, a candidate microbicide, and methylcellulose placebo vaginal gels among HIV-positive women and men in Durban, South Africa.. AIDS Res Ther.

[8] Rosen RK, Morrow KM, Carballo-Dieguez A, Mantell JE, Hoffman S (2008). Acceptability of tenofovir gel as a vaginal microbicide among women in a phase I trial: a mixed-methods study.. J Womens Health.

[9] Morrow KM, Ruiz MS (2008). Assessing microbicide acceptability: A comprehensive and integrated approach.. AIDS Behav.

[10] Skoler-Karpoff S, Ramjee G, Ahmed K, Altini L, Plegianos MG (2008). Efficacy of Carraguard® for prevention of HIV infection among women in South Africa: a randomized, placebo-controlled trial.. Lancet.

[11] Coetzee N, Blanchard K, Ellertson C, Hoosen A, Friedland B (2001). Acceptability and feasibility of Micralax® applicators and of methylcellulose placebo for large-scale clinical trials of vaginal microbicides.. AIDS.

[12] McLean C, van de Wijgert J, Jones H, Karon J, Chaikummao S (2010). HIV-1 Genital shedding and safety of Carraguard use by HIV-1-infected women: A randomized, controlled, cross-over Trial in Thailand.. AIDS.

[13] Whitehead SJ, Kilmarx PH, Blanchard K, Manopaiboon C, Chaikimmao S (2006). Acceptability of Carraguard vaginal gel use among Thai couples.. AIDS.

[14] Jones HE, Chaikummao S, van de Wijgert JH, Friedland BA, Manopaiboon C (2009). Acceptability of a carrageenan-based candidate vaginal microbicide and matching placebo: Findings from a Phase II safety trial among women in Chiang Rai, Thailand.. J Women's Health.

[15] Kilmarx PH, van de Wijgert JH, Chaikummao S, Jones HE, Limakarnjanarat K (2006). Safety and acceptability of the candidate microbicide Carraguard in Thai women: Findings from a phase II clinical trial.. J Acquir Immun Defic Syndr.

[16] Kilmarx PH, Blanchard K, Chaikummao S, Friedland BA, Srivirojana N (2008). A randomized, placebo-controlled trial to assess the safety and acceptability of use of Carraguard vaginal gel by heterosexual couples in Thailand.. Sex Transm Dis.

[17] Morrow K, Rosen R, Richter L, Emans A, Forbes A (2003). The acceptability of an investigational vaginal microbicide, PRO 2000 gel, among women in a phase I clinical trial.. J Women's Health.

[18] Braunstein S, van de Wijgert J (2005). Preferences and practices related to vaginal lubrication: Implications for microbicide acceptability and clinical testing.. J Women's Health.

[19] Lai BE, Xie YQ, Lavine ML, Szeri AJ, Owen DH (2008). Dilution of microbicide gels with vaginal fluid and semen simulants: Effect on rheological properties and coating flow.. J Pharm Sci.

[20] Owen DH, Peters JJ, Kieweg SL, Geonotti AR, Schnaare RL (2007). Biophysical analysis of prototype microbicide gels.. J Pharm Sci.

[21] Mauck C, Rosenberg Z, van Damme L, for the International Working Group on Microbicides (2001). Recommendations for the clinical development of topical microbicides – an update.. AIDS.

[22] Abdool Karim Q, Abdool Karim SS, Frohlich JA, Grobler AC, Baxter C (2010). Effectiveness and safety of tenofovir gel, an antiretroviral microbicide, for the prevention of HIV infection in women.. Science.

