# Supplementary material for: Acceptability of Carraguard Vaginal Microbicide Gel among HIV-Infected Women in Chiang Rai, Thailand
Source: PLoS One. 2011 Sep 7;6(9):e14831. doi: 10.1371/journal.pone.0014831 (PMC3168444; doi:10.1371/journal.pone.0014831)
Supplement: Protocol S1 — Trial protocol. (0.48 MB DOC) [file pone.0014831.s001.doc]

**A Randomized, Controlled, Double-Blind, Cross-Over Trial of Safety, Effect on Genital Tract HIV Shedding, and Acceptability of Vaginal Use of Carraguard by HIV-Infected Women**

6/28/06

Version 5.0

Table of Contents

[1 Study summary 3](#__RefHeading___Toc139333215)

[2 Study personnel and funding 3](#__RefHeading___Toc139333216)

[3 Background 3](#__RefHeading___Toc139333217)

[4 Objectives 3](#__RefHeading___Toc139333218)

[5 Methods 3](#__RefHeading___Toc139333219)

[5.1 Overview 3](#__RefHeading___Toc139333220)

[5.2 Design 3](#__RefHeading___Toc139333221)

[5.3 Study products 3](#__RefHeading___Toc139333222)

[5.4 Study arms 3](#__RefHeading___Toc139333223)

[5.5 Study arm sequences 3](#__RefHeading___Toc139333224)

[5.6 Sample size 3](#__RefHeading___Toc139333225)

[5.7 Study site 3](#__RefHeading___Toc139333226)

[5.8 Study population 3](#__RefHeading___Toc139333227)

[5.9 Eligibility criteria 3](#__RefHeading___Toc139333228)

[5.10 Study recruitment 3](#__RefHeading___Toc139333229)

[5.11 Screening visit #1 3](#__RefHeading___Toc139333230)

[5.12 HIV evaluation 3](#__RefHeading___Toc139333231)

[5.13 Screening visit #2 3](#__RefHeading___Toc139333232)

[5.14 Partner HIV testing and informed consent 3](#__RefHeading___Toc139333233)

[5.15 Enrollment visit 3](#__RefHeading___Toc139333234)

[5.16 Randomization and dispensing of study products 3](#__RefHeading___Toc139333235)

[5.17 Product administration plan 3](#__RefHeading___Toc139333236)

[5.18 Follow-up visits 3](#__RefHeading___Toc139333237)

[5.19 Specimen collection and laboratory studies 3](#__RefHeading___Toc139333238)

[5.20 Reimbursement, risks, and benefits 3](#__RefHeading___Toc139333239)

[5.21 Data collection and management 3](#__RefHeading___Toc139333240)

[5.22 Data analysis 3](#__RefHeading___Toc139333241)

[5.23 Study timeline 3](#__RefHeading___Toc139333242)

[5.24 Training 3](#__RefHeading___Toc139333243)

[6 Human subjects review, informed consent, and confidentiality 3](#__RefHeading___Toc139333244)

[7 Adverse events 3](#__RefHeading___Toc139333245)

[8 Serious adverse events 3](#__RefHeading___Toc139333246)

[9 Study monitoring 3](#__RefHeading___Toc139333247)

[10 Product interruption criteria 3](#__RefHeading___Toc139333248)

[11 Trial closure considerations 3](#__RefHeading___Toc139333249)

[12 Protocol amendments 3](#__RefHeading___Toc139333250)

[13 Appendices 3](#__RefHeading___Toc139333251)

[14 Study forms 3](#__RefHeading___Toc139333252)

# Study summary

**Design**

Randomized, controlled, double-blinded, cross-over trial

**Sample size**

60 HIV-infected women

**Population**

HIV-infected women, recruited from general medical clinics, family planning clinics, groups/organizations working with persons living with AIDS, and if necessary, public advertising. Women must plan to be abstinent or in a seroconcordant relationship with only one partner for the study period.

**Study objectives**

To assess product safety, effect on genital tract HIV shedding, and product acceptability with vaginal use of Carraguard gel among HIV-infected women.

**Study products**

Carraguard gel

Placebo (methyl cellulose gel)

**Study arms**

Carraguard (A)

Placebo (B)

No product (C)

**Study plan**

Women will be screened for eligibility, which will include two screening visits, HIV, and tuberculosis testing, and an HIV clinical evaluation. If a woman has a husband or steady sex partner, he will need to come to the clinic for HIV testing to confirm that he is HIV-infected (and therefore not at risk for HIV infection related to the woman’s study participation) and to provide informed consent, because he may also be exposed to the study product, prior to her enrollment. Informed consent will be obtained. This study includes three study arms: Carraguard, placebo, and a no product arm. During the study, each woman will participate in each study arm, and she will be randomized to one of six study arm sequences (see section 5.5). At the enrollment visit, a pelvic examination will be conducted. During the examination, a baseline cervico-vaginal lavage (CVL) HIV sample and vaginal swab sample will be collected to quantify genital tract HIV, and a baseline colposcopic examination will be conducted to evaluate the mucosal epithelium. After the pelvic examination is conducted, the woman will be randomized to a study arm sequence. When receives a product, she will insert the gel vaginally as directed while still at the clinic. The participant and the research staff will be blinded regarding which gel the participant is using. Fifteen minutes after the product is inserted, a second pelvic examination will be conducted, 2 CVLs and vaginal swab samples will be collected, and a repeat colposcopic examination will be conducted to assess any acute effects of product exposure. If it is determined that one CVL is adequate to assess acute effects, only one CVL will be collected after the gel application at each baseline visit for the duration of the study. Participants who do not use a product will also undergo a second pelvic examination with CVL, vaginal swab and colposcopy at 15 minutes. During each study arm, the participant will use the designated product (or no product) for one week, beginning 3-5 days after the end of her menstrual period. She will have a total of two follow-up visits at day 7 and day 14 (1 and 8 days after discontinuing product use). Women and their partners will be counseled regarding the use of condoms and will be given condoms at no cost, to prevent transmission of different viral strains between partners. Women will be asked to return to the clinic for a pelvic examination with CVL and vaginal swab sample collection for HIV and colposcopy. On day 14 (~7 days after discontinuing product use), another pelvic examination with CVL and vaginal swab for HIV will be conducted. Women will then be asked to wait until 3-5 days after the end of the next menstrual cycle to return to clinic for the first visit in the next arm of their study arm sequence baseline study visit). Data from the baseline study visit will also be used for analysis of day 28 delayed effects or potential carry-over effects from the previous arm. A questionnaire regarding product use, acceptability, and adherence will be administered at the first follow-up visit (day 7) of each product arm. All women will participate in each study arm. The three study visits and data collection described above will be repeated for each of the 3 study arms.

**Benefits**

- HIV medical care referral
- Screening for tuberculosis
- If participant is positive for tuberculosis, she will be referred for tuberculosis preventive therapy and reimbursed for the 9 month tuberculosis prevention program
- One-time subsidy up to 1,000 baht for HIV medical consultation, laboratory, radiology and pharmacy fees at Chiang Rai Hospital
- Laboratory testing: CD4 count, viral load, and complete blood count
- Diagnosis and treatment of reproductive tract infections (RTI)
- Safer sex counseling and free condoms

**Risks**

- Vaginal irritation/lesions, including ulcerations, due to potential toxicity of the study product(s)
- Discomfort and/or minimal vaginal bleeding during and/or after pelvic examination
- Increased risk of RTIs if the participant is sexually active and if the study product increases this risk
- Bruising and discomfort at phlebotomy site
- Psychological stress, including embarrassment during the pelvic examination
- Stress on relationship(s) if a sexually transmitted disease is diagnosed
- Inconvenience of multiple clinic visits

# Study personnel and funding

This study proposal is the result of an established collaboration between the Population Council, the Thailand Ministry of Health and US CDC Collaboration, the Chiang Rai Public Health Office, and Chiang Rai Hospital. The Thailand Ministry of Health and US CDC Collaboration is funded by the Division of HIV/AIDS Prevention at the Centers for Disease Control and Prevention.

**The Chiang Rai Health Club**

The Chiang Rai Health Club was established in 1991 as a field research station by the Thailand Ministry of Health and US CDC Collaboration, a joint activity of the Thai Ministry of Public Health and the U.S. Centers for Disease Control and Prevention. The Chiang Rai Health Club of the Thailand Ministry of Health and US CDC Collaboration has conducted evaluations of risk perception and willingness to participate in a microbicide trial and a phase I clinical trial of PC-503, a microbicide closely related to Carraguard (1). A phase II trial of Carraguard™ in HIV seronegative women (Population Council IRB-approved protocol 271; CDC approved protocol 2485) and a phase II trial of HIV negative couples (Population Council IRB-approved protocol 270; CDC approved protocol 2968) are in progress at the Chiang Rai Health Club. The Chiang Rai Microbicide Research Community Advisory Group, a local advisory group with representatives of local organizations and community members, was formed to assist in the development of microbicidal studies in Chiang Rai and to assure that the research agenda is responsive to the needs of the community.

**The Population Council**

The Population Council’s microbicides program is a collaborative effort between the Population Council’s Center for Biomedical Research (CBR) and the International Programs Division (IPD). Scientists at CBR conduct basic research on disease transmission and test a variety of potential microbicides – both contraceptive and non-contraceptive – *in vitro* and in animal models. Cell culture systems, developed for documenting and quantifying the process of viral transmission and lymphocyte adhesion, have facilitated CBR’s study of product ability to block transmission of HIV and other sexually transmitted pathogens. Compounds showing sufficient promise at CBR are tested in human trials conducted by researchers in IPD. IPD clinical research has focused primarily on several carrageenan formulations, most recently, Carraguard™.

**Principal investigators**

Catherine McLean,

Principal Investigator, CDC

Medical Epidemiologist

Division of STD Prevention

National Center for HIV, STD, and TB Prevention

Centers for Disease Control and Prevention

1600 Clifton Road, MS E-02

Atlanta, GA 30324

cvm9@cdc.gov

tel: (404)639-8467

fax: (404)639-8610

Jordan Tappero,

GAP/TUC Director

On-site Principal Investigator, CDC

Thailand Ministry of Health and US CDC Collaboration

Thai, international and express mail:

DMS Building 6, MOPH

Tivanon Road, Nonthaburi

11000 Thailand

U.S. Mail (domestic rates):

Box 68 CDC/HIV

APO AP 96546

Tel. 66-2-591-8358

Fax 66-2-591-5443

Mobile phone (66 1) 755 9011

Taweesap Siraprapasiri

Principal Investigator

Adjunct Deputy Director, TUC

Thailand Ministry of Health and US CDC Collaboration

Nonthaburi, Thailand

DMS 6 Building; Ministry of Public Health

Tivanon Road; Nonthaburi 11000, Thailand

auf7@CDC.gov

Tel. 66-2-591-8358

fax: (66-2)591-5443

Janneke van deWijgert

Population Council Consultant

International Antiviral Therapy Evaluation Center

Academic Medical Center, University of Amsterdam

Meibergdreef 9

PO Box 22700

1100 DE Amsterdam

Tel (mobile): +31-6-23848893

Fax:+31-20-6918821

Philip Guest

Principal Investigator, Population Council

Senior Associate and Country Representative

Population Council

P.O. Box 138

Pratunam Post Office

Bangkok, 10409 THAILAND

[Philip@popcouncil.th.com](mailto:Philip@popcouncil.th.com)

Tel: 66-2-251-4766

Fax: 66-2-255-5513

Supaporn Chaikummao

Medical Research Coordinator

Chiang Rai On-site Principal Investigator

Thailand Ministry of Health and US CDC Collaboration

Thai, international and express mail:

DMS Building 6, MOPH

Tivanon Road, Nonthaburi

11000 Thailand

U.S. Mail (domestic rates):

Box 68 CDC/HIV

APO AP 96546

Email: szk0@CDC.gov

Tel. 66-1-8504343 cell

Fax. 66-2-591-5443

Co-primary investigators

Thepnarumit Medtanavyn, Provincial Chief Medical Officer

Jullapong Achalapong, Staff, Department of Obstetrics and Gynecology

**Co-investigators**

Peter Kilmarx,

CDC, Global AIDS program

Director, BOTUSA ProjectDirector, BOTUSA Project

Office tel: 267-301-696; 267-303-532

Cell phone (permanent): 267-713-17878

Residence (permanent): 267-312-300; 267-303-931

Fax: 267-581-697

E-mail: pbk4@cdc.gov

*Botswana and international mail:*

BOTUSA ProjectBOTUSA Project

PO Box 90

Gaborone, Botswana

Sara Whitehead

Chief, Chiang Rai Field Station

Thailand Ministry of Health and US CDC Collaboration

Thai, international and express mail:

DMS Building 6, MOPH

Tivanon Road, Nonthaburi

11000 Thailand

U.S. Mail (domestic rates):

Box 68 CDC/HIV

APO AP 96546

Email: szk0@CDC.gov

Tel. 66-1-8504343 cell

Fax. 66-2-591-5443

Thanyanan Chaowanachan, Chief, Laboratory Section - Study Laboratory Coordinator

Philip Mock, Chief, Data/Computer Unit - Study Data Manager

Lies Bollen**,** Medical Officer, Chiang Rai Section

Chomnad Manopaiboon, Behavioral Scientist

Chiang Rai Public Health Office

Mayuree Wankrairoj, Chief, STD/AIDS Section

Chiang Rai Hospital

Renu Srismith, Director

Somboonsak Yanpaisarn, Chief, Department of Obstetrics and Gynecology

Wat Uthaivoravit, Chief, Department of Preventive Medicine

Pacharee Kantipong, Chief, Department of Medicine

Paisit Witwatwongwana, Staff, Department of Obstetrics and Gynecology

Centers for Disease Control and Prevention

National Center for HIV, STD, and TB Prevention, Atlanta

*Division of STD Prevention*

Lauri Markowitz, Chief, Epidemiology Research Section

Maya R. Sternberg, Mathematical Statistician, Data and Statistics Branch - Study Statistician

National Center for Infectious Diseases

*Division of AIDS, STD and TB Laboratory Research*

Clyde Hart, NCID, Chief, Retrovirology section

Tammy Evans-Strickfaden, Microbiologist

Cheng Chen, Microbiologist

Elizabeth Unger, Virologist

Population Council New York

Sarah Braunstein, Population Council Study Coordinator (sbraunstein@popcouncil.org)

Population Council Bangkok

Nucharee Srivirojana, Study Monitor ([nuch@popcouncil.th.com](mailto:nuch@popcouncil.th.com))

Nattaya Boonpakdee, Study Community Liaison ([nattaya@popcouncil.th.com](mailto:nattaya@popcouncil.th.com))

**Consultants**

*Division of HIV/AIDS Prevention--Surveillance & Epidemiology, Epidemiology Branch*

Lynn Paxton, Chief, Transmission Section

John Karon

Statistical consultant (located in Alburquerque, New Mexico USA)

Employed by Emergint Corp, Louisville, KY, as a subcontractor for

Northrup-Grumman Corp as contractor for

Centers for Disease Control and Prevention

National Center for HIV, STD, and TB Prevention

[JKaron@earthlink.net](mailto:JKaron@earthlink.net)

505.342.5639

# Background

There are approximately 33.6 million people living with HIV/AIDS in the world today, and 95% live in the developing world. Over 70% of all HIV-1 infections in adults worldwide are acquired through heterosexual intercourse (1). In South and Southeast Asia, there are over 6 million HIV-infected persons living with HIV/AIDS (1), and most HIV transmission in the region, including in Thailand, is heterosexual (2).

Microbicides are substances that can be used vaginally or rectally during sexual intercourse to prevent infection, and would provide women with the potential to protect themselves and their sexual partners from HIV and other sexually transmitted infections (STIs). Microbicides may be used in combination with condoms to increase protection or, in situations in which condoms are not being used, as primary protection. There are several mechanisms of action through which microbicides may work, including by forming a barrier between pathogen and target epithelium (i.e. Carraguard), preventing replication of pathogens (i.e. nucleoside reverse transcriptase inhibitors), or by killing, or immobilizing, pathogens (i.e. nonoxynol-9). There are currently more than 50 potential microbicides under development.

Current recommendations to decrease HIV transmission, including mutual monogamy, condom use, and STI treatment, are difficult for some women to adopt. Mutual monogamy requires a sex partner’s cooperation, condom use is a male-controlled method, and STI treatment is often difficult to achieve because many STIs are asymptomatic.

Prevention methods that do not require partner cooperation, such as microbicides or vaccines, are needed, particularly in regions where the epidemic is primarily heterosexual.

In many of the communities in which a microbicide is likely to be used, there are high rates of HIV infection. Many women who choose to use a microbicide may not know they are HIV-infected. Others may be aware that they have HIV and wish to use a microbicide to avoid infection with other STIs, or to protect their partners from HIV infection. Depending upon their mechanism of action, microbicidal products could either increase or decrease HIV shedding in women who are HIV-infected and may impact HIV transmission. Therefore, data on the effect of a potential microbicide in HIV-infected women are important.

Carraguard is a leading microbicide candidate and has been shown to prevent mucosal transmission in laboratory studies (3). The active pharmaceutical ingredient in Carraguard is a sulfated polysaccharide mixture of *lambda-* and *kappa-*carrageenan (FMC, PDR98-15) derived from the seaweed species *Chondrus crispus*, with a continuum molecular weight of 150,000 to 10 million. In the Carraguard gel formulation, the carrageenan mixture is dissolved in purified water with 0.1% p-hydroxybenzoic methyl ester added as a preservative. Phosphate-buffered saline (PBS) and hydrochloric acid are used to adjust the pH to 7.

In a recently developed HIV mouse system, Carraguard has been shown to block transmission of HIV across the vaginal epithelium. Carraguard has also been shown to inhibit infection by
*N. gonorrhoeae*, HSV-2, and HPV *in vitro* and *in vivo* (3). Vaginal formulations of Carraguard are highly effective in protecting mice from HSV-2 infection. In fact, Carraguard is more effective in protecting mice from HSV-2 infection than either of the over-the-counter nonoxynol-9-containing vaginal spermicides, Gynol II or KY Plus(3), or other microbicides under development. Studies conducted in Rhesus Macaque monkeys showed that PC-503, a sister formulation to Carraguard with the same active ingredient, *lambda*-carrageenan, is as effective in preventing SIV infection as 3% nonoxynol-9 gel or 12% nonoxynol-9 foam over-the-counter products (3). *In vitro* laboratory studies have also shown that carrageenan did not inhibit or enhance the growth of *Lactobacillus acidophilus*, the most common of the naturally occurring vaginal flora. Carraguard™ is a non-contraceptive gel: it has not been shown to kill or immobilize sperm *in vitro*.

There is no evidence that Carraguard would increase susceptibility to STIs or HIV. In fact, all the evidence points in the opposite direction. The available in vitro and animal data cited in the protocol suggest that Carraguard will be protective against HIV and other STIs.

The reference to the theoretical increased risk of acquiring STIs is included in the consent because of the hypothetical risk of increased epithelial disruption (ulceration/abrasion/fissure), and if that occurs, the possible increased susceptibility to HIV/STIs (2). Because these women are already HIV positive, they are not susceptible to HIV.

Epithelial disruption is one of our primary endpoints, and a critical endpoint to assess in the evaluation of a new microbicidal agent (16). Studies of other vaginal microbicides (unrelated to Carraguard) have found increases in epithelial disruption with use in HIV negative women (e.g. Col-1492), potentially increasing their susceptibility to HIV.

There are no data indicating that patients enrolled in this or other microbicide studies are more susceptible to new strains of HIV. In addition, many of the participants in this study will not be sexually active, and those who are will be counseled regarding correct and regular condom use.

Reproductive toxicology tests in rats have been conducted using Carraguard™. Reproductive toxicology tests in rats consist of two segments: Segment I studies evaluate the effect of a product on fertility and early embryonic development up to implantation stage, and Segment II studies on implantation up to gestation. In Segment I studies, Carraguard™ had no adverse effect on fertility, implantation and behavior, even at high doses (final report pending).

In this study, there is an extremely small chance of gel use in very early pregnancy (women start using gel just after their period, and are using the gel for only one week in each cycle), and the segment I rat studies cover that period. The Population Council will seek an exemption for Segment II studies, based on Segment I results for Carraguard™, and existing reproductive toxicology results for carrageenan. The Population Council is asking for an exemption for segment II testing because Carraguard is not absorbed. However, if the US FDA does not grant an exemption, Segment II studies in rats will be conducted concurrently with the Phase III trial which will be conducted in Botswana and S. Africa, beginning late 2002- 2003. Notably, the FDA has asked the Population Council to allow HIV-negative pregnant women to continue to use gel during their pregnancy in the Phase III trial (indicating a low level of concern about teratogenicity of Carraguard).

There are no data indicating that patients enrolled in this or other microbicide studies are more susceptible to new strains of HIV.

Carrageenans have been used extensively in the food, pharmaceutical and cosmetics industries as lubricants, emulsifiers, and stabilizing agents. The use of seaweed extracts for medicinal purposes can be traced back hundreds of years (4). The compounds are on the U.S. FDA’s Agenerally recognized as safe“ (GRAS) list and are deemed to be safe for human consumption and topical application (4). Toxicology studies on the use of carrageenans as vaginal gels include the *in vivo* tests in rabbits conducted by NAmSA®, which indicated that they were not irritants to the vaginal mucosal tissue of the rabbit (3).

The Population Council has completed two Phase I clinical studies of carrageenan formulations in humans. The Council received IND approval from the U.S. Food and Drug Administration (FDA) for the *iota*-carrageenan-based microbicide known as PC-213 in 1996 (3). The results of multi-site Phase I trials of PC-213 conducted through the Council’s International Committee on Contraceptive Research (ICCR) network showed no signs of irritation among women using the product once a day for seven days (5). Work in the laboratory during this clinical testing however, showed that *lambda*-carrageenan (PC-503), another type of carrageenan, was more promising, and the Council received IND approval from the FDA for PC-503 in the fall of 1997. In 1998, the Population Council completed a multi-site Phase I safety trial on PC-503, using a protocol almost identical to that used for the PC-213 trials. These trials were conducted at four sites through the Council’s ICCR network (Australia, Chile, Dominican Republic, and the United States) and at the Chiang Rai Health Club of the Thailand Ministry of Health and US CDC Collaboration in Chiang Rai, Thailand. None of the women who applied the product once daily for seven days experienced any significant irritation (6).

Further refinement of the carrageenan gel in the laboratory resulted in a third, yet more promising, formulation 3% lambda- and kappa-carrageenan, referred to as Carraguard. In addition, the Population Council filed a trademark application to use the name Carraguard for its carrageenan-based microbicides. The Council’s lead microbicide, Carraguard, has since been referred to as Carraguard.

Currently, a multi-center, Phase I/II safety and acceptability study of Carraguard is being conducted in two South African sites and at the Chiang Rai Health Club of the Thailand Ministry of Health and US CDC Collaboration in Chiang Rai, Thailand. The complete Phase I/II study will enroll a total of approximately 565 HIV-negative women attending family planning and general health clinics at the three sites (~200 women at each of the South African sites and 165 at the Thai site). Half of the cohort at each site is randomized to Carraguard gel and half to the placebo gel (2.5% methyl cellulose). Women are instructed to use gel vaginally three times per week (approximately one dose every other day) during the entire trial period (6-12 months per woman), regardless of whether they engage in vaginal intercourse. Women are also instructed to insert gel no more than one hour prior to vaginal intercourse each time that they have intercourse, with no maximum limit on the number of sex acts.

In South Africa, the first 15 women enrolled at each site (n’30 total) provided initial safety information, and a Data Safety and Monitoring Board (DSMB) reviewed data from an interim analysis after 100 woman-years of follow-up. The unblinded results of these analyses are on file at the Population Council. They did not warrant any changes to the main study, and follow-up of the remainder of the cohort is currently continuing as planned. In Thailand, a DSMB reviewed data from two interim analyses, one after 25 woman-months of follow-up and the second after 75 woman-years. Both times the DSMB recommended continuing the trial as planned. Data collection in both trials will be completed in November 2001.

While there is considerable variability in cervico-vaginal HIV “shedding,” there is an association between plasma HIV RNA and cervico-vaginal HIV levels with higher plasma HIV viral load associated with increased cervico-vaginal “shedding QUOTE(7;8).” Similarly, there is an association between HIV genital tract shedding and CD4 counts, with lower CD4 counts associated with higher amounts of “shedding” QUOTE{ ADDIN REFMAN #\11\05ê\19\01\00\00\00\03(5)\00\03\00\1Dl:\5Clink\5Ccvm9\5Crefman\5Cnewrefman\03\00\0211 John, Nduati, et al. 1997 11 /id\00 \00 (9). Given that lower CD4 counts and higher plasma viral loads are associated with increased “shedding,” persons on effective antiretroviral therapy for HIV are less likely to have detectable levels of cervico-vaginal HIV. While pilot programs exist to make antiretrovirals available to some patients, currently the great majority of HIV-infected people in Thailand do not receive any antiretroviral therapy.

HIV viral shedding is also associated with local inflammation due to sexually transmitted infections (STIs) and cervical atypia. Several studies suggest that STIs and reproductive-tract inflammation may increase levels of HIV-1 shedding in genital secretions and may, therefore, lead to increased infectiousness and transmission of HIV-1 (10). One study has shown that seminal HIV-1 RNA concentrations were eight times higher in men with urethritis than in men without urethritis QUOTE(11). In this study, treatment for urethritis resulted in substantially lower concentrations of urethral HIV-1 RNA. These data suggest that inflammation increases HIV RNA shedding and may increase transmission of HIV-1 infection.

The association between cervico-vaginal HIV viral shedding and hormonal fluctuations during the menstrual cycle and pregnancy is unclear. A cross-sectional study in HIV-infected women attending STD clinics in Mombasa, Kenya found that cervico-vaginal HIV-1 shedding is associated with hormonal contraceptive use (13)QUOTE(7). However, a subsequent study found no pattern of cervico-vaginal HIV shedding with phase of the menstrual cycle, nor with serum estradiol or progesterone levels QUOTE{ ADDIN REFMAN #\11\05ê\19\01\00\00\00\03(8)\00\03\00\1Dl:\5Clink\5Ccvm9\5Crefman\5Cnewrefman\03\00\0214#Mostad, Jackson, et al. 1998 14 /id\00#\00 (12).

HIV genital tract Ashedding” has been associated with perinatal transmission of HIV. In a randomized, placebo-controlled study of short course antenatal zidovudine, researchers found that the presence of cervico-vaginal HIV RNA is an independent risk factor for perinatal HIV transmission. In this study, women with quantifiable HIV RNA levels in cervico-vaginal samples at 38 weeks gestation had a 15% transmission risk while women without quantifiable HIV RNA levels had a 1% transmission risk (13). Other studies have confirmed these findings QUOTE{ ADDIN REFMAN #\11\05ê\19\01\00\00\00\03(2)\00\03\00\1Dl:\5Clink\5Ccvm9\5Crefman\5Cnewrefman\03\00\0215#Panther, Tucker, et al. 2000 15 /id\00#\00 (14).

These data suggest that cervico-vaginal HIV shedding is an important, potentially modifiable risk factor in the sexual and vertical transmission of HIV-1 and may be an important surrogate marker of infectiousness. Determining how potential microbicides affect genital HIV shedding is a critical step in vaginal microbicide product development (15).

The Thai family planning program (developed ~30 years ago with strong support from the Population Council) makes contraceptive options available at low or no cost to women in Thailand, and family planning is widely acceptable. (See reference: Chamratrithirong A, Kamnuansilpa P, Knodel J. Contraceptive practice and fertility in Thailand: results of the Third Contraceptive Prevalence Survey. Stud Fam Plann 1986;17:278-87.)

The Third Contraceptive Prevalence Survey in Thailand was conducted in 1984. Results indicate a continuation of the rapid rise in contraceptive use among married couples that has been taking place over the past 15 years. Prevalence of family planning use is approaching that of economically advanced countries. Sterilization is now the most common method, although a fairly broad range of other methods is also widely used. Only modest levels of unmet need for contraception for either limiting family size or spacing children now exist. Fertility rates have fallen since the previous survey, done three years earlier, but to a lesser extent than would be expected from the increased use of contraceptives. Family size preferences are concentrated at small family sizes. A comparison between the Buddhist majority and Moslem minority, made possible through a special sample design, reveals substantial differences between the two groups. Contraceptive use is lower and fertility levels and preferences are higher among Muslims than among Buddhists.

# Objectives

The objectives of the proposed study are to assess the effect of daily vaginal use of Carraguard gel by HIV-infected women on

- Safety
- Genital tract HIV shedding
- Product acceptability

Endpoints will be measured at the first follow-up visit (on study day 7), at the second follow-up visit (on study day 14, 7 days after discontinuing product use), and the next baseline visit (on study day 28, 21 days after discontinuing product use) and will be compared with the baseline assessment on day 0.

Acute effects of the product will be measured 15 minutes after the first application (enrollment visit).

Objective #1: To assess safety

Safety of daily product use will be determined by:

1. Symptoms of irritation
2. Effect on vaginal flora
3. Vaginal epithelial disruption as determined by naked eye inspection (and colposcopic inspection on day 7), according to established guidelines (16), and
4. Other adverse effects (which may include UTI or other unforeseen problems)

Objective #2: To assess effect on genital tract HIV viral load

Genital tract HIV will be measured using samples collected by CVL and vaginal swab, and viral load determinations will be performed using the COBAS AMPLICOR HIV-1 MONITOR v.1.5 Test Kit. In addition, levels of infectious virus will be determined using the multinuclear activation of a galactosidase indicator (MAGI) assay that identifies infectious cell-free HIV-1 in vaginal secretions.

Objective #3: To assess product acceptability

Women will be asked to complete an interview-administered questionnaire regarding acceptability of the product once each study arm at the first follow-up visit (day 7), after using the product daily for 7 days.

# Methods

## Overview

Prior to enrollment in the study, women will have two screening visits and an HIV medical evaluation at Chiang Rai Hospital to determine whether she is eligible to enroll in the study. In addition, women will ask their husband or steady male partner to be HIV tested to confirm that he is HIV-infected. He will need to sign an informed consent, because he may also be exposed to the study product, prior to enrollment. If eligible, women will be asked to provide informed consent prior to study enrollment. Women will participate in three study arms (two product, one no-product) and will be randomized to one of 6 possible study arm sequences (see section 5.5). Women will be asked to come to the clinic to begin each study arm approximately 3-5 days after the end of her menstrual period. Women using depo-provera will be followed on a 28 day schedule. At the first baseline visit (enrollment), a pelvic examination will be conducted, baseline CVL and vaginal swab samples will be collected for cervico-vaginal HIV viral load, and a colposcopic examination to assess the vaginal epithelium will be conducted. Immediately following the first examination and specimen collection, women who have been randomized to a product arm, will vaginally insert the first dose of the product, using the applicator as instructed. The participant will use the first product (or no product if in the no-product arm) in the sequence to which she is randomized. Approximately 15 minutes after she has inserted the product, the HIV CVL, swab and colposcopic examination will be repeated to assess acute effects of the product. If the arm of the sequence to which she is randomized is the arm in which no product is used, she will follow the same study visit schedule and will follow the same study steps as the other participants. Women will wait in the clinic 15 minutes, and then will be examined and have the HIV CVL, swab, and colposcopic examination repeated. Women who are assigned to a product arm will apply the product at home once daily for a total of seven days (Table 1). On study day 7 (following 7 days of daily product use), participants (irregardless of whether they are in a product or no product arm) will return to the clinic for another pelvic examination when cervico-vaginal HIV viral load measurements (using CVL and vaginal swabs) and colposcopic examinations will be repeated. At the second follow-up visit on day 14 (7 days after discontinuing product use), participants (whether they are in a product or no product arm) will return to the clinic for another pelvic examination when cervico-vaginal HIV viral load measurements (using CVL and vaginal swabs) will be collected. Women will then return to the clinic approximately 3-5 days after the end of the next menstrual period (day 28 for women using depo-provera) and, at that time, after a three week washout period, will begin the next study arm in her study arm sequence (See Table 1).

## Design

Randomized, controlled, double-blind, three arm, cross-over trial

## Study products

The Population Council will supply Carraguard and its matching placebo in boxes of pre-packaged tube-shaped Microlax applicators which contain 7 mL of the product and dispense 4 mL of the product upon self-administration. The product and placebo will be manufactured according to the Population Council’s specifications and in accordance with FDA guidelines. Shipping of study product to the study site will occur according to a shipping Standard Operating Procedure (SOP). Shipments are labeled with FDA-mandated labels, indicating that they contain a ANew Drug Limited by Federal Law (USA)” and are for AInvestigational Use Only.@ They also contain detailed information about the contents of the shipment and relevant contact information.

**Composition of Carraguard**

Carraguard is the Population Council’s leading microbicide candidate. The active pharmaceutical ingredient (API) in Carraguard is a sulfated polysaccharide mixture of mostly *lambda-* with a smaller amount of *kappa-*carrageenan (FMC, PDR98-15), both derived from the seaweed species *Chondrus crispus*. The molecular weight is between 150,000 and 10 million. In the Carraguard gel formulation, the carrageenan mixture is dissolved in purified water with 0.1% p-hydroxybenzoic methyl ester added as a preservative. Phosphate-buffered saline (PBS) and hydrochloric acid are used to adjust the pH to 7.

**Composition of placebo**

The placebo product is a gel that contains 2.5% methyl cellulose dissolved in purified water with 0.1% p-hydroxybenzoic methyl ester added as a preservative. PBS and hydrochloric acid are used to adjust the pH to 7. The placebo looks, feels, smells and tastes the same as Carraguard; both are clear gels. Methyl cellulose is neither spermicidal or microbicidal. Methyl cellulose gel was selected as the placebo gel based on investigations in the HSV-2 mouse model. Mice were pretreated with either methyl cellulose, K-Y Jelly, or Carbopol, compared with the control group who received nothing or PBS. Mice were then inoculated with 10^4^ pfu HSV-2, a dose previously shown to infect half of the untreated mice. K-Y Jelly and Carbopol exhibited some slight inhibitory effects on HSV-2 infection, and methyl cellulose gel had no effect compared to treatment with PBS or no pretreatment (16). Preliminary evidence also shows that methyl cellulose gel has no effect against *N. gonorrhoeae* in a mouse model (Zacharopolous VR, unpublished data), nor against human papilloma virus (HPV) in a mouse model (M Howett, unpublished data). Finally, like *lambda*-carrageenan, methyl cellulose neither kills nor immobilizes sperm (Phillips DM, unpublished data).

## Study arms

- Carraguard (A)
- Placebo (Methyl cellulose gel) (B):
- No product (C)

## Study arm sequences

- A-B-C (1)
- A-C-B (2)
- B-A-C (3)
- B-C-A (4)
- C-B-A (5)
- C-A-B (6)

## Sample size

The two primary hypotheses in this study are that Carraguard is 1) different from placebo and 2) different from no product. A primary outcome is cervico-vaginal HIV shedding measured after 7 days of daily product use. The power calculation is based on ensuring adequate power for testing these two hypotheses with this outcome with an overall significance level of 0.05. We used a 3x3 Williams design to determine power/sample size (see graph below). The Williams design is a cross-over design balanced for first carry-over effects when testing products used in sequence and will provide minimum variance unbiased estimates for the treatment effect comparisons in the presence of first order carry-over effects. It is not known whether the washout period used in this study design will be sufficient to assume no carry-over effects, hence a Williams design was employed to ensure unbiased minimum variance estimates. If there are no carry-over effects (i.e. the power will be higher than indicated) the power calculations for this design are conservative.

The null hypotheses for these primary hypotheses are: 1) No difference between Carraguard and no product and 2) no difference between Carraguard and placebo.

In order to carry out the power calculations the within subject variability needs to be specified, as well as an effect size, defined here as the average difference between a pair of treatments. Estimates of the underlying variability of genital tract HIV shedding are based on preliminary data from the CDC laboratory studies (unpublished), where the vaginal viral load was measured weekly throughout the menstrual cycle (i.e. for three weeks after menses) for 45 HIV-infected women. Based on a one-way repeated measures ANOVA, the within standard deviation of cervico-vaginal HIV shedding on the log scale was estimated as 0.66.

The following plot shows the power to detect a statistically significant difference between a pair of treatments for varying effect sizes with an overall sample size of 30 or 50, at a significance level of 0.025 (based on the Bonferroni adjustment for the two planned pairwise comparisons, for an overall Type I error rate of 0.05). A horizontal line indicates 80% power. The effect size is based on the difference between a pair of treatment means on the log scale. Thus the plot shows that with a sample size of 30 and a within standard deviation of 0.6, there is at least 80% power to detect a difference between a pair of treatment means (on the log scale) of 0.55 or higher. On the other hand, with a sample size of 50 and a standard deviation of 0.6, there is at least 80% power to detect a difference between a pair of treatment means (on the log scale) of 0.43 or higher. The estimates of power would be higher if no carryover effect truly exists. Sixty women will be enrolled to account for participants lost to follow-up and participants who may start antiretroviral therapy and so there is an equal number of women in each study sequence.

## Study site


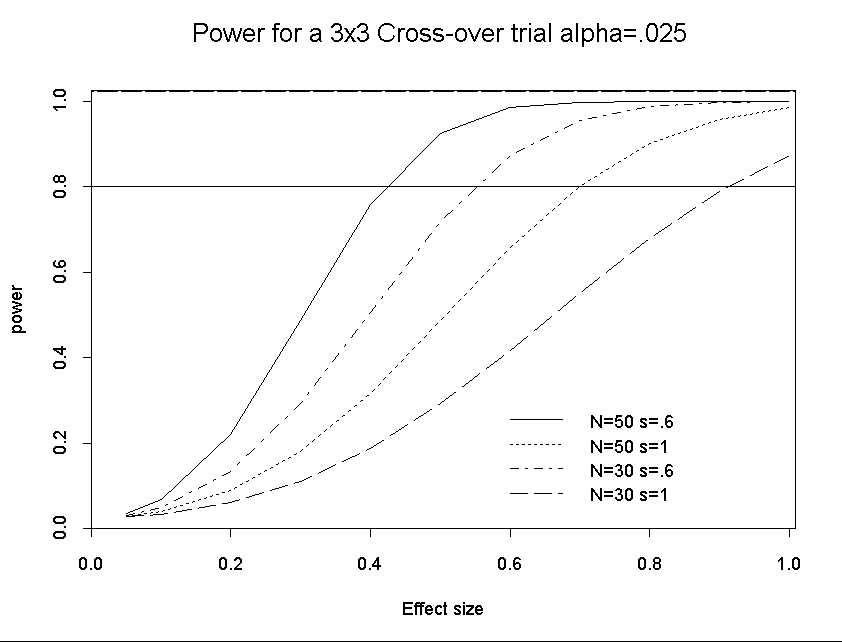


The study site is the Chiang Rai Health Center of the Thailand Ministry of Health and US CDC Collaboration (TUC)(described in more detail in section 2), located in Chiang Rai, Thailand. The TUC enjoys a strong working relationship with the Provincial Public Health Office and local public clinic sites including Chiang Rai Hospital.  TUC staff in Chiang Rai includes an international medical epidemiologist, a research coordinator, 5 research nurses, 4 research assistants, a laboratory scientist, and a data entry clerk, each of whom will spend half of their time working on this study while it is being conducted.  There is also strong administrative, laboratory, and data management support from the main office in Bangkok.  The group has extensive experience in performing cross-sectional and cohort studies including participant recruitment and follow-up, obtaining informed consent, interviewing, counseling, and referral, as well as performing pelvic examination, colposcopy, cervicography, and CVL. Clinic visits in this study will take place at the Chiang Rai Health Center (formerly the STD clinic) where the current phase I and II studies are being conducted and where the TUC has dedicated space for a research office, waiting room, laboratory, two pelvic exam rooms, and four counseling rooms.

## Study population

The study population will consist of 60 HIV-infected women, recruited from general medical and family planning clinics, persons living with AIDS groups, and if necessary, from public advertising. The study sample will not include minors, pregnant women, mentally retarded, or mentally disabled women.

## Eligibility criteria

Women will be eligible to participate in the study if they fulfill all of the following inclusion criteria:

- Age 18-50 years
- Plan to stay in the Chiang Rai area for at least four months
- HIV-infected, confirmed by Elisa and Western Blot tests
- Either has 1) no current sex partner and is planning to be abstinent for the study duration, *or* 2) has only one, HIV-infected sexual partner^1^ who is 18 years or older and is willing to give informed consent for confirmatory HIV testing and for the participant’s enrollment in the study. Women who have a steady partner/husband who she will not see during the study period or with whom she is not sexually active will not be required to bring their partner to the clinic for HIV testing and informed consent.
- Willing and able to give informed consent
- Willing and able to comply with the study protocol, including being tested for HIV and undergoing repeated pelvic and colposcopic examinations
- Willing to have male partner asked for informed consent because he will be exposed to study product
- Regular menstrual cycles (defined as occurring every 3-5 weeks, lasting 3-5 days) for the prior 3 months; if have amenorrhea or if using depo-provera, participant must have no reported history of vaginal bleeding for the previous 3 months
- CD4 count < 500^2^
- Not currently taking antiretroviral medications^3^
- Documented Class I or Class II (“atypical cells seen, usually caused by inflammation”) pap smear at screening for study participation
- In good health as determined by medical history, physical examination and results of any laboratory screening test, and the discretion of the clinical staff
- Able to achieve a score of 80% or better on true-false test of key study concepts. If women score less than 80% the first time they take the test, they may repeat the test at least one day later

^1^We are including this criterion to minimize any potential risk of HIV transmission from study participants to a sexual partner.

^2^We are including this criteria because women with CD4 counts greater than 500 are much less likely to have cervical-vaginal HIV shedding.

^3^We are including this criterion because women who are on antiretroviral therapy are much less likely to have cervical-vaginal HIV shedding. Women who begin antiretroviral therapy while enrolled in the trial will be allowed to continue to participate in the trial.

Women will be excluded from the study if any of the following **exclusion criteria** apply:

- CD4 count <50
- Pregnancy or desire to become pregnant in the next 3-4 months
- Delivery, miscarriage, or abortion within six weeks prior to study enrollment
- History of surgery on external genitalia, vagina or cervix in the month prior to study enrollment
- Existence of a clinically detectable genital abnormality, specifically warts or a congenital abnormality
- History of nonmenstrual vaginal bleeding with intercourse in past one month
- Current use of tampons, diaphragms, sponges, douching, or other intravaginal products. Women who are willing to abstain from using these products during the study will be included in the study.
- Concurrent participation in another trial of a vaginal product
- History of sensitivity or allergy to latex products (including gloves)
- Presence of epithelial disruption of the labia or genital mucosa visible to the naked eye at enrollment^1^
- Positive test for gonorrhea, chlamydial infection, trichomoniasis, or evidence of untreated syphilis (see section 5.19). Participants with positive tests must be treated and have a negative test of cure to be eligible for enrollment.^1^
- Positive tests for candidiasis or bacterial vaginosis (BV) *and* symptoms of vaginitis. Women may be enrolled after treatment if they are asymptomatic or have negative tests. Asymptomatic participants with positive tests for candidiasis or BV may be enrolled.

^1^If an RTI is diagnosed, treatment will be given, and the participant will be allowed to begin the study once she has completed treatment for the RTI and has had a test of cure. A clinically significant ulcer will be treated empirically for syphilis and chancroid (unless it is clearly herpetic), and she will be eligible for enrollment once the ulcer has resolved. Women with other persistent abnormal signs, such as vaginal or cervical discharge, despite treatment of the RTI will be eligible. Participants who test positive for an RTI must have a negative RTI test collected less than one month prior to her enrollment in the study. HPV detection will not be a criterion for exclusion if the women meet the Pap criteria for enrollment.

## Study recruitment

All HIV-infected women attending the recruiting clinics will be asked if they are interested in learning more about an opportunity to participate in a study of a vaginal product that may reduce HIV transmission. The following issues will then be reviewed:

- Study objectives
- Eligibility and exclusion criteria
- Study protocol requirements, including having a clinical evaluation three times per month for 3 months, inserting a vaginal product daily for 7 consecutive days each month, and having their partner tested for HIV and giving his informed consent (because of his exposure to the product).

If a woman is interested in participating, she will be given additional information regarding the study, and a screening appointment will be scheduled.

## Screening visit #1

The first screening visit at approximately day (–30) will include the following (see Tables 2 and 3):

- Interviewer-administered eligibility checklist
- Explanation of the study goals, methods, risks, and benefits
- Informed consent for screening
- Pre-test HIV counseling
- Blood draw for rapid plasma regain (RPR) test, HIV, and CD4 count
- HIV medical care referral (based on self-reported HIV status)
- Resources for additional information regarding the study

Information will be provided regarding study risks, benefits, and resources if participants have further questions regarding the study. At this time, women will also have an opportunity to discuss questions with a counselor in private. A screening interview will be conducted, and blood will be drawn for HIV, syphilis, and CD4 count. The woman will then be referred to Chiang Rai Hospital for an HIV medical care evaluation. Women will be taken to Chiang Rai Hospital by a study nurse with a referral letter and CRH Assessment form which will be carried in a sealed envelope.

If a woman is eligible and agrees to be screened for the study, a brief comprehension test will be administered after she has provided informed consent for screening and before she provides informed consent for enrollment to confirm that she understands the purpose of the study and the potential risks and benefits. If she scores over 80% on the comprehension test, written informed consent for the screening evaluation will be obtained. If she scores less than 80%, she will not be enrolled. She will be allowed to retake the comprehension test once, but it must be at least one day after the initial test.

## HIV evaluation

The HIV medical care evaluation will be conducted at The Department of Medicine at Chiang Rai Hospital and includes the following:

- HIV medical care review
- Assessment of eligibility for treatment with antiretroviral therapy
- Evaluation for tuberculosis preventive therapy

Participants will be referred to Chiang Rai Hospital for evaluation and treatment. Actual medicals costs incurred at Chiang Rai Hospital, including medical consultation, laboratory, radiology, and pharmacy fees, will be reimbursed by the study to the women for up to 1,000 Baht (approximately USD 22). In addition, the study will provide reimbursement for the nine-month tuberculosis prevention program for women who test positive for tuberculosis. Medicines for treating HIV infection will not be offered to participants as a part of this study.

Relatively few people with HIV/AIDS in Thailand have access to antiretroviral therapy (ART) through limited programs sponsored by the Ministry of Public Health. Currently the largest such program provides ART to approximately 2,500 people nationwide out of the estimated 600,000 people with HIV/AIDS in Thailand. Access to these programs is controlled by district-level committees. This situation is evolving with ART becoming more generally available. ART will not be offered to participants as a part of this study. Offering such therapy as a benefit of study participation could be an unduly coercive incentive to enrollment, forcing women who would not otherwise do so to use the study gel. In addition, provision of ART requires long-term commitment and rigorous attention to adherence, response to treatment, emergence of resistance, and monitoring for side effects, beyond the scope of this limited research protocol. During the study, staff will facilitate access to available ART programs for study participants in collaboration with the Chiang Rai Hospital Department of Medicine. If women are started on antiretroviral therapy during the initial HIV evaluation, they will not be eligible for the study. Participants will not be prevented or discouraged in any way from initiating antiretroviral therapy once they enroll, if it becomes available to them. If this occurs, participants will continue to follow the same procedures as those who are not on antiretroviral therapy; This procedure will be re-evaluated if the situation changes in Thailand, e.g., if access to antiretroviral therapy is expanded to make the medications available to most patients. Both the Community Advisory Group in Chiang Rai and the Thai Ethical Review Council support our plan.

The data collected from a woman who begins antiretroviral therapy will be analyzed and described separately from the other study participants because antiretroviral therapy will affect the amount of cervico-vaginal HIV shedding.

## Screening visit #2

The second screening visit, approximately day (–14) will include the following (see Tables 2 and 3):

- Review of study goals, methods, risks, and benefits
- Post-test HIV counseling
- Reproductive tract infection (RTI) symptoms, signs, and prevention counseling
- Family planning counseling
- Screening clinician form
- Urine pregnancy test
- Pelvic examination
- Pap smear
- Sample collection for RTIs
- Resources for additional information and/or complications and suspected side effects during the study

The study counselor will provide all women with intensive counseling regarding HIV and STI risk communication, partner HIV testing, condom use, and safer sex practices. Counseling will emphasize that abstinence or mutual monogamy is recommended. Male condom use for every sexual encounter will be strongly recommended to prevent transmission of different viral strains between partners, and proper condom use will be explained and demonstrated. Latex condoms, without N-9 lubrication, will be provided at no charge, and women will be asked to use only study condoms (to avoid possible confounding that might result from the use of N-9-lubricated condoms). STI signs, symptoms, and risk factors will be reviewed, and women will be counseled to continue their current method of family planning. Because the desire to become pregnant during the study period is an exclusion criterion for this trial, sexually active women not currently using a family planning method will be encouraged to adopt one, and will be referred to local family planning services if needed. If a woman does get pregnant during the study, she will be asked to immediately discontinue study product use. She will be referred for medical care and will be followed until the end of the study arm, then discontinued from the study, because pregnancy is an exclusion criterion for the study. If a woman meets study enrollment criteria, she will be asked to return to the clinic for enrollment 3-5 days after her next menstrual period has ended (or in 2 weeks [i.e., 4 weeks after her first screening visit] if she is using depo-provera). Sexually active women will be asked to abstain from unprotected vaginal intercourse for the 24 hours prior to each clinic visit. Women will also be asked not to douche or wash their genitals on study visit days.

For women for whom there is > 1 month between first and second screening visits, the eligibility checklist will be completed again at the second screening to confirm that her eligibility has not changed.

**Management of RTIs**

Prior to study enrollment, pelvic examination, Pap smear, and RTI screening will be conducted, and RTIs will be treated according to the latest Thai (1997) and CDC (1998) guidelines.

Participants will receive treatment for all curable RTIs free of charge. Treatment of genital ulcer disease will be syndromic and will include treatment for chancroid and syphilis, unless the ulcer disease is highly suspicious for herpes. Treatment of cervical and vaginal infection will be guided by RTI test results. At all visits where speculum examination is performed, vaginal pool specimen pH, and gram stain for BV and yeast will be conducted on samples collected from the posterior fornix. For women with symptoms or abnormal signs, stat gram stain, trich in-pouch, and KOH whiff test will be conducted, and results of the pH, KOH whiff, and gram stain will be available immediately following the examination. Women with symptomatic bacterial vaginosis (BV) or symptomatic candidiasis will therefore be treated at the same study visit. Results for the trichomonas culture results will be available within several days and for *C. trachomatis* and *N. gonorrhoeae* will be available within two weeks of the study visit, and women with positive results will be treated either at the next regularly scheduled follow-up visit or, if the study staff feel that it is warranted, the woman would be contacted (with her permission and in a manner that preserves confidentiality) to come to the clinic for an unscheduled visit. Study staff may treat severe cervicitis or symptoms of pelvic inflammatory disease syndromically at the study visit, it they feel that this is necessary to prevent sequelae.

During the study, genital ulcer disease will be treated syndromically including adequate therapy for curable etiologies (syphilis and chancroid). Amplicor genital ulcer PCR is not approved by the US or Thai FDA and is not routinely available in Thailand. Because PCR genital ulcer disease (GUD) specimen testing will be conducted after the study is completed, participants will be asked if they would like to be contacted and informed of their genital ulcer PCR test results (if this type of testing is conducted during the study). All women will have a pap smear as part of the study screening process and will be advised at enrollment to have regular annual pap smears. HPV testing will be conducted after the study is completed; Clinical management is not affected by these results, and results will not be provided to patients. Women with curable STIs (gonorrhea, chlamydia, trichomoniasis, or syphilis) will be given a card (with a code to indicate the infection type) to give to her partner(s), if she chooses, so that he/they can come to the clinic for testing and treatment. Participants’ partners will be treated for STIs free of charge.

Women with any curable RTI must be effectively treated prior to enrollment. Women may be enrolled after treatment, and women with treated chlamydia infection or gonorrhea may be enrolled after having a test of cure approximately four weeks after completing therapy and less than four weeks before enrollment. Women with syphilis may be enrolled once they are free of signs and symptoms of syphilis and if their RPR titer has declined at least four-fold following treatment. Women with treated trichomoniasis may be enrolled if there is no evidence of trichomoniasis on repeat trichomonas InPouch wet-mount & culture examination. Women with other persistent abnormal signs, such as vaginal or cervical discharge, despite treatment of identified RTI, may be enrolled. Women with symptoms of vaginal infection may be enrolled only if their current stat laboratory evaluation is negative for BV, candidiasis, and trichomoniasis. Women with asymptomatic BV or candidiasis may be enrolled. Women with laboratory evidence of or recent treatment for BV or candidiasis may be enrolled once asymptomatic. Women with persistent genital epithelial disruption on naked eye examination or colposcopy will not be enrolled.

All women will also be asked to give contact information so that they can be located in the event that they do not return to the clinic for a scheduled study visit. The study staff will explain to the women that attendance at scheduled follow-up visits is important, especially in the event that one of her laboratory tests shows a positive diagnosis of an STI for which she could be treated. The contact information, however, will not become part of the case record forms and will be kept in a locked filing cabinet, separate from the forms

## Partner HIV testing and informed consent

If the woman has a sex partner, he will be required to come to the clinic for an HIV test and to give his written, informed consent for her participation in the study prior to her enrollment because of his potential exposure to the study product. If she will not see or does not plan to be sexually active with her husband or steady sex partner during the study period, he does not need to come to the clinic for HIV testing and informed consent. Women will receive extensive counseling prior to study enrollment regarding the risk of HIV transmission from her to any sex partner. During the counseling sessions, she will be strongly encouraged to use condoms, and condom use will be reviewed. During the study screening and pre-enrollment process, women will be counseled regarding the importance of remaining abstinent or using condoms if she is sexually active during the study. If she initially plans not to be sexually active with her steady partner/husband during the study and her situation changes, she will be asked to bring him to the clinic for informed consent and HIV testing.

If women who had thought they would not see their steady sex partner or husband during the study report that they have become sexually active or if there is evidence that they are sexually active, they will be asked to bring him to the clinic for informed consent and HIV testing. Women who are not able to be compliant with the study protocol, especially if others may be at risk as a result, may be discontinued from the study at any time based upon the investigators’ discretion.

## Enrollment visit

After the woman has completed two screening visits and had an HIV care evaluation at Chiang Rai Hospital, she will come to the clinic for her first study visit ideally, 3-5 days after the end of her menstrual cycle. (This visit may occur up to 10 days following the end of her menstrual cycle if her day 14 visit is not expected to coincide with the estimated start of her next menstrual cycle.) The enrollment visit is the baseline visit of the first study arm. During the enrollment visit, the following will take place (see Tables 2 and 3):

- Review of STI results and treatment confirmation
- Confirm partner’s informed consent
- Informed consent for enrollment
- Enrollment form
- Interviewer-administered enrollment interview
- Urine pregnancy test
- pH, Gram Stain,
- Collection of blood sample for plasma viral load testing, HSV 1 and 2 antibody testing
- Baseline pelvic and colposcopic examinations
- Baseline CVL and vaginal swab sample collection for HIV cervico-vaginal viral load
- Clinician study form
- Study arm sequence randomization
- Product administration (if a product arm)
- Second pelvic and colposcopic examinations (to assess acute effects of the product on the vaginal epithelium)
- Second CVL and vaginal swab sample collection for cervico-vaginal HIV viral load (to assess acute effects of the product on cervico-vaginal HIV viral load)

The details of the study will be discussed again before enrollment, and women will be asked to sign an informed consent for study enrollment. Consent from the woman’s husband/steady partner is required because of his potential exposure to the gel, and this consent must be obtained prior to the participant’s enrollment in the study. He will be able to come to the clinic anytime after the woman has had her first screening visit and her enrollment visit to be tested and give his informed consent. A research nurse will administer the enrollment interview that includes questions concerning the participant’s reproductive health and sexual practices.

A pelvic examination will be conducted in which samples for RTI testing and cervico-vaginal HIV viral load will be collected, and colposcopy will be conducted. A rapid semen detection test will be conducted on the CVL to determine if the CVL is contaminated with semen which could effect the HIV viral measurement in the CVL, resulting in an inaccurate measurement of the study participant’s cervico-vaginal HIV viral load. If the CVL is contaminated with semen, this CVL and corresponding swab will be discarded, and the enrollment visit will be rescheduled within 48 hours.

Patients in whom the rapid semen detection test is positive will receive extensive counseling regarding risk of HIV transmission to sex partners and strongly encouraged to use condoms or remain abstinent during the study. Regardless of reported partner status, all study participants will be counseled to use condoms consistently throughout the study.

After the baseline examination and clinical specimen collection, women will be randomized to a study arm sequence. If the first arm of her study sequence is a product arm, she will be given instructions on how to insert the product and, while in the examination room, will vaginally insert the first applicator of gel. She will wait either in the examination room or in the waiting room. After approximately 15 minutes, another pelvic examination will be conducted, cervico-vaginal HIV viral load samples including 2 vaginal swabs and 2 CVL (unless further study indicates that only one is necessary) will be collected, and colposcopy will be done. Neither the woman nor the research team will know which of the two products she is using.

If the first study arm to which the participant is randomized is the arm of the study in which noproduct is used, she will also wait at the clinic for 15 minutes and will have a repeat pelvic examination with cervico-vaginal HIV sample collection and colposcopy.

Women assigned to a product arm will be instructed to insert the gel vaginally once daily for the following 6 days. Women will be given 7 applicators (one extra) to take home.

## Randomization and dispensing of study products

This study is a cross-over design in which each woman will participate in each of the 3 study arms. Once enrolled in the study, women will be randomized to one of six study arm sequences. Before the study begins, a pseudo-random number generator, available as part of the SPSS software package, will be used to generate a randomization scheme, assigning women to one of the 6 sequences, beginning with Carraguard, placebo, or a no product arm.

Individual envelopes will be prepared for each woman in the study, each containing a unique study identification number. Upon enrollment in the study, each woman will receive the next sequentially numbered envelope. The envelope will contain a code indicating to the study staff her study arm sequence, or the order in which she will participate in each of the three arms: Carraguard, placebo or no product. The study staff will refer to this code when distributing the study products to the participants. Additional participants added to maintain the number of evaluable subjects will be added in groups of six, randomized to one of the six study sequences.

Study gels will be packaged in boxes of eight applicators, with different colored seals indicating Carraguard or placebo. Once randomized, a participant will be considered enrolled in the study and will be included in the intention-to-treat analyses; only women who are started on antiretroviral therapy will be excluded from the intention-to-treat analysis.

## Product administration plan

Women will be instructed to insert one dose of gel, either Carraguard or placebo, vaginally every evening for the six days following the baseline visit. Women will be asked not to use other vaginal products, during the study period. Lubricated latex condoms with reservoir tips, and which are not lubricated with N-9, will be provided to limit any potential exposure to N-9. All participants will be informed that the study gel is to be used only vaginally, and that it is not designed for oral or rectal use.

## Follow-up visits

At follow-up visits the participant will be asked about the following:

- Occurrence of any medical problem or adverse event
- Sexual behavior and condom use
- Use of other vaginal products
- Study product use (day 7 only)
- Product acceptability (day 7 of second gel arm only)

At any time during the study, if a participant has questions, concerns, or unusual symptoms, she will be asked to return to the clinic promptly to meet with a study nurse or to have a clinical evaluation. If participants report for unscheduled visits, an evaluation of the existing problem will be undertaken and any necessary treatment will be provided. At all study visits, a rapid semen detection test will be conducted using a vaginal swab. If contaminated with semen, the visit will be rescheduled for 24-48 hours later.

Follow-up visit #1 (day 7):

The first follow-up visit (day 7) (for those assigned to a product arm, this visit follows seven days of product use) includes the following (See Tables 2 and 3):

- Interviewer-administered questionnaire concerning reproductive health, sexual practices, symptoms, experience using the product (Acceptability questionnaire). This is administered on day 7 of second gel arm only.
- Pelvic and colposcopic examinations
- Vaginal pool pH, Gram stain specimen collection and testing
- Sample for cervico-vaginal HIV viral load
- Clinician study form

Participants will be asked to bring all used and unused applicators to the clinic at each follow-up visit at which time they will be counted and logged. Women will be asked to return used applicators in individual sealable plastic bags, provided to the participants. Women will be asked if they tried to squeeze out all of the contents of the applicator, or if they chose not to use all of the product. At each Follow-up visit #1, the investigator will estimate study product and condom use based on a count of the used and remaining unused applicators and on an interview with the participant.

If participants have used study gel for less than 6 of the previous 7 days, they will be asked to use the study gel for up to 3 more days for a maximum of 6 days and return to the clinic at that time to complete the first follow-up visit.

During the pelvic examination and before the cervico-vaginal HIV viral load sample collection, a rapid semen detection test will be conducted using a vaginal swab. If contaminated with semen, the visit will be rescheduled for 24-48 hours later.

Follow-up visit #2 (day 14):

The second follow-up visit (day 14) (for those assigned to a product arm, this visit occurs7 days after the woman has stopped using the product), includes the following (See Tables 1-3):

- Pelvic examination
- Vaginal pool pH, and Gram stain specimen collection
- HPV testing
- Sample for cervico-vaginal HIV viral load
- Clinician study form

Women will then be asked to return to the clinic to begin the next study arm 3-5 days after the next menstrual period has ended.

Baseline visit:

The baseline visit will be the first visit of the new study arm. Women using depo provera will be placed on a regular 28 day schedule, so that day 0 will be approximately day 28 of the preceding cycle. In the event that a participant begins the study and she is unable to start her second or third study arms on time, we will allow the woman to start her next arm the following month. The Baseline visit, similar to the enrollment study visit, will include the following (See Tables 2 and 3):

- Interviewer-administered enrollment interview
- Collection of blood sample for plasma HIV viral load testing, HSV 1 and 2 antibody testing
- Baseline pelvic and colposcopic examinations
- Baseline sample collection for RTIs
- Baseline sample collection for cervico-vaginal HIV viral load
- Product administration (if a product arm)
- Second pelvic and colposcopic examinations (to assess acute effects of the product on the vaginal epithelium)
- Second sample collection for cervico-vaginal HIV viral load (to assess acute effects of the product on cervico-vaginal HIV viral load)
- Clinician study form

(The second examination and sample collection will not be conducted at the final study visit, day 28 of the third study arm).

## Specimen collection and laboratory studies

The following samples will be collected for HIV and RTI testing when indicated in the protocol (see Table 2):

1. Blood (venipucture) for HIV and CD4 count will be conducted once at the beginning of the study.
2. HIV plasma viral load will be conducted at each baseline, a total of three times
3. Cervical/endocervical swab will be used to collect a pap smear specimen once at the second screening visit
4. Cervical swab for HPV testing at screening visit #2 and at each of the day 14 visits, a total of 4 swabs
5. Swabs (2) of the posterior fornix and endocervix for diagnosis of sexually transmitted infections will be collected at the second screening visit and day 28 of the 3^rd^ arm, and repeated if signs or symptoms of STI develop during the study period (Total swabs’ 4).
6. Swabs (2) of the posterior fornix for Gram stain and pH, will be collected at each study visit in which an exam is conducted (Total swabs’22 )
7. Syphilis serologic testing will be conducted once at the beginning of the study unless a genital ulcer or other suggestive symptoms are identified
8. CVL specimens will be collected during each study visit in which a clinical exam is conducted, to measure cervico-vaginal HIV viral load by introducing 5 ml of PBS into the vagina and collecting the pooled fluid in the posterior vaginal fornix. A total of 16 CVLs will be obtained from each woman during the study.
9. Two vaginal swabs will be used during each pelvic exam during the study to collect cervico-vaginal HIV viral load samples (20 swabs). At the second exam (“acute effects” assessment) on day 0 of each arm, four swabs will be used to collect the cervico-vaginal HIV viral load samples (12 swabs)(Total swabs’ 32).

9) Urine pregnancy test at screening #2 and each baseline visit (4 urine samples)

10) Genital ulcer sample collection (if genital ulcer present clinically)

11) A swab will be collected at each study visit to test for semen contamination (11 swabs)

12) HSV-1 and HSV-2 serology on stored blood specimens from all baseline clinic visits (Total of 3 visits per woman - 180 stored specimens total).

13) Cytokine testing on stored CVL specimens from all baseline clinic visits and all day 7 visits (Total of 6 visits per woman - 360 stored specimens total).

All laboratory specimens from each visit will be identified by the same specimen number using pre-printed waterproof stickers. The specimen number will be recorded in the corresponding visit case record form and in a specimen logbook.

The majority of testing will be done at the TUC laboratory in Bangkok. Selected specialized HIV and STI testing will be conducted at CDC in Atlanta, and trichomoniasis testing and limited testing for candida will be conducted at Chiang Rai Health Center.

For each of the indications below, the following tests will be conducted:

*Trichomoniasis vaginalis*: The InPouch culture test (BioMed Diagnostics, San Jose, California, USA) from a swab of the posterior fornix to detect *T. vaginalis.* With the InPouch system more than 90% of positive cultures are identified within the first 24 hours. *In vitro* testing has shown that Carraguard could interfere with trichomonas growth at 48 but not at 24 hours (17).

*Candida sp*: Gram stain will be used to detect *Candida sp.*

*Bacterial vaginalis:* Gram stain will be used to assess the vaginal flora for BV, using the Nugent criteria. For women with symptoms, Amsel criteria will also be used.

*N. gonorrhoeae and C. trachomatis*: A COBAS Amplicor polymerase chain reaction (PCR) (Amplicor, Branchburg, NJ, USA) *N. gonorrhoeae* and *C. trachomatis* test will be conducted from an endocervical specimen. A modified collection and processing procedure using a chlamydia transport medium and a PBS wash will be used to eliminate all inhibition from the Amplicor test (17).

Syphilis: A non-treponemal, rapid plasma reagin (RPR) test (MacroVue RPR Card Test, Becton Dickinson Microbiology Systems, Cockeysville, Maryland, USA) will be used for the serological detection of syphilis infection. A reactive syphilis test will be confirmed by the *Treponema pallidum* particle-agglutination assay (TPPA; TPPA Reagents, Fujirebio Inc., Tokyo).

Etiology of genital ulcer disease will be determined by a genital ulcer, multiplex polymerase chain reaction (PCR) assay (Cheng Chen's lab, CDC) for the presence of *H. ducreyi*, *T. pallidum*, and Herpes simplex virus (HSV).

Human papillomavirus (HPV): Cervical swabs will be tested for HPV using L1 consensus PCR with the PGMY09/PG MY11 primer set. The resulting PCR products will be typed using a line blot assay (developed by Roche Molecular Systems, Alameda, CA).

Cervical cytology: Cytology will be performed by pathologists at Chiang Rai Hospital laboratory.

HIV: an enzyme immunoassay (EIA, Genetic Systems HIV-1/HIV-2 EIA, Genetic Systems Corp., Redmond, Washington) will be used for screening. A Western blot (WB, NovaPath HIV-1 Immunoblot, BioRad Diagnostics Group, Hercules, California) will be used for confirmation.

CD4 count: Lymphocyte phenotyping will be performed on venous samples collected in EDTA collection tubes using the FACScan flow cytometer (Becton Dickinson Immunocytometry Systems, San Jose, CA, USA) and the standard six-tube, two-color monoclonal antibody panel (Becton Dickinson).

HIV plasma viral load: HIV RNA levels in the plasma will be determined by using the Amplicor HIV-1 Monitor Test, version 1.5 (Roche Diagnostic Systems, Branchburg, NJ), a quantitative reverse-transcriptase PCR assay. The lower quantification limit for the standard Monitor assay is 400 copies/ml, and the lower quantification limit for the Ultrasensitive Monitor assay is 50 copies/mL.

Cervico-vaginal HIV viral load measurements: Five milliliter (PBS) CVL samples will be examined for blood by visual inspection and with the use of a Multistix-8 SG Urine Strip (Bayer Inc., Elkhart, IN) that measures whole blood and hemoglobin. If blood is present by Multistix-8, hemoglobin concentrations will be used to calculate blood volumes in CVL.

The cervico-vaginal samples will be separated into cellular and cell-free fractions by low speed centrifugation (400 x g). A 1.0 ml aliquot of CVL supernatant will be used to calculate the HIV-1 RNA copies per total CVL. Cell-free, virion-associated HIV-1 RNA in CVL will be quantified using the Roche Amplicor Monitor HIV-1 ver.1.5 kit (Roche Diagnostic Systems, Branchburg, NJ), per the manufacturer’s protocol, with the exception that viral pellets will be obtained by centrifugation (1 x 10^5^ x g) for 60 minutes and a NucliSense wash will be used to extract the viral RNA prior to running the amplicor test. The CVL cell pellets will be used to test for cellular replication of HIV-1 in vaginal secretions using a semiquantitative test for spliced HIV-1 mRNA. Infectious virus in CVL will be measured using a recently developed modification of the TZM-bl assay that detects low levels of infectious HIV-1 in female genital secretions.

It is known that Carraguard inhibits the RNA Amplicor test used for the cervico-vaginal supernatant specimens collected during the acute effects assessment if Carraguard is present in high enough quantities. The lab protocol includes a dilution step that may be sufficient; however, if the concentration of Carraguard is too high for RNA testing to be valid, we will replace the RNA Amplicor test with an HIV p24 antigen test of specimens.

**Semen Detection:** Testing to be done on a vaginal swab specimen collected according to manufacture’s instructions (Abacus Diagnostics). If contaminated with semen, the woman will be rescheduled for another CVL and swab collection for within 24-48 hours.

HSV 1 and HSV 2 serology: testing will be done according to manufacturers instructions on stored serology specimens.

Cytokine testing: Cytokine testing will include IL-1 beta and TNF alpha (and may include a broader range of specific cytokines, if resources permit) and are measured with a kit Elisa from R&D Systems (Human IL-1 beta Quantikine(r) kit; Human TNF-alpha Quantikine(r) kit).

## Reimbursement, risks, and benefits

Reimbursement

Participants in this study will be given 300 Baht (approximately USD 6.50) per scheduled visit. This sum is intended to reimburse participants for transportation to and from the clinic and other potential costs associated with study participation, such as lost time from work and child care. Participants will be given 50 Baht (approximately USD 1.10) for any unscheduled visits.

Benefits

- Initial HIV medical consultation, laboratory, radiology, and pharmacy fees at Chiang Rai Hospital (Subsidized up to 1000 Baht).
- Diagnosis and treatment of reproductive tract infections
- Referral for tuberculosis preventive therapy.
- Reimbursement for the cost of 9 month tuberculosis prevention program
- One-time free laboratory testing: complete blood count and lymphocyte subset enumeration
- Participants’ partners will be treated for STIs free of charge
- Safer sex counseling
- Free condom distribution and counseling

Risks

- Vaginal lesions, including ulcerations, due to the potential toxicity of the study products
- Discomfort and/or minimal vaginal bleeding during or after pelvic examination
- Bruising and discomfort at the venipuncture site
- Psychological risks include stress on relationships if an STI is diagnosed
- Possibility of increased susceptibility to reproductive tract infections (RTIs) due to study product use
- Possibility of increased HIV infectiousness due to study product use
- Possibility of embarrassment during the pelvic examination

## Data collection and management

Case record forms are to be completed in black or blue ballpoint pen at the time of the participant visits. Information on the case record forms will be reviewed three times for completeness and clarity: by the local study staff after each study visit, at monitoring visits, and prior to data entry. The study staff will check the questionnaires and physical exam forms for completeness before the participants leave the clinic to ensure that any answers to questions missed or left blank can be ascertained. The study monitors will be responsible for assuring that the forms are properly completed and retraining of the study staff will be undertaken if necessary. Accuracy of data entry will be checked by the data analysis team who will inspect and confirm apparently aberrant responses. The laboratory scientist will complete the laboratory forms and keep duplicate records in the laboratory. Coding of adverse events and concomitant medication will be done by an experienced study team member. Case record forms will be used to create a database at the TUC. All data will be double entered and validated in Chiang Rai .

All forms will have two copies. One copy will be kept at the study site, and the original will be sent to the Population Council. To correct errors, a single line will be placed through the mistake, a date, and the initial of the person making the change will be included. No whiteout or other obscuring method will be used.

Questions emerging during the different data management steps will be sent by the Population Council data manager to the investigators. The database will be edited once the questions are resolved. Once the data has been entered and cleaned, the database will be sent to CDC.

Data analysis will be led by a CDC statistician in close collaboration with the Population Council Data Manager and the Principal Investigators from The Population Council, TUC, and CDC.

## Data analysis

Objective 1: Product safety

Primary endpoints include epithelial disruption based upon colposcopic and naked-eye exams, vaginal flora disturbances, self-reported symptoms, and acquisition of RTIs. A random effects general linear model will be used which includes parameters for subject effects, treatment effects, period effects and first-order carry-over effects.

##

Objective 2: Genital shedding of HIV

Analyses will be based on a log transformation of the genital tract HIV viral load data, as suggested by preliminary data on genital tract HIV shedding from the CDC laboratory studies (unpublished). A random effects linear model will be used to analyze the data collected at the first follow-up visit, 7 days after daily product use. This model includes parameters for subject effects, treatment effects, period effects and first-order carry-over effects (defined as carry-over effects from the previous period). The subject effect will be treated as a random effect with a normal distribution. The random error term associated with a specific period and subject within a given sequence will be assumed to have a normal distribution with a compound symmetric (uniform) covariance structure. The chosen model assumes no sequence effect, which is reasonable given subjects are being randomized to each sequence. In addition, we assume no treatment by period interaction (i.e. the effect of genital tract HIV shedding for each treatment does not depend on the period it is administered), which seems to be a reasonable assumption for this study. Wash-out periods of three weeks between each treatment are expected to lessen the chances of observing a significant carryover effect. However, since little is known about the effects of these treatments on genital tract HIV viral load, the possibility of a carry-over effect will be tested. In the absence of carry-over effects, this will be dropped from the model and we can estimate the direct effect of the treatments on genital tract HIV viral load.

Objective 3: Product acceptability

Results from the Acceptability questionnaires will be tabulated, and associations between baseline demographic and sexual behavior characteristics and indicators of acceptability will be explored.

Weekly reports on enrollment and follow-up statistics will be distributed by Chiang Rai Health Club staff to the study team to assess trends.

The definition of noncompliance is less than 6 days of study gel use reported at the first follow-up (7 day ) visit. Women who do not meet this criterion will be asked to use gel for up to 3 more days, for a maximum of 6 days. For the analysis, women who use study gel 6 of 9 days or more will be included in the per protocol analysis and those who do not meet this criterion will be included in the intention to treat analysis.

We will have a statistician review the mucosal safety data after the first 25 women have completed the first gel use arm. We will look at the differences in epithelial disruption (includes abrasions, ulcers, and fissures) from baseline to the first follow-up visit (day 7) and compare these differences between the 3 study arms. We will stop enrollment in the study and form a DMC to review the data if there is a difference between groups that is significant at the p<0.001 level, a standard stopping rule for this type of trial (Reference: Geller, N and Pocock, S. Interim Analyses in Randomized Clinical trials: Ramifications and Guidelines for Practitioners. Biometrics 43 1987 213-223).

Study participants will be provided with information regarding how to contact the Chiang Rai Health Center for study results and their blinding code when results are available. Results of the final analysis will be presented to the Chiang Rai Advisory Committee, and at local and international scientific meetings. We also plan to publish the results in the international scientific literature.

## Study timeline

Month 1-3: Training and begin enrollment

Month 4-6: Complete enrollment

Month 7-9: Complete follow-up

Month 10-12: Complete analysis

Start date: March 2003

## Training

Several weeks before beginning study enrollment, additional training will be conducted on the study procedure with the study staff in Chiang Rai.

# Human subjects review, informed consent, and confidentiality

The protocol requires that written informed consent be obtained for all study participants. The readability of the written informed consent forms will not exceed the 8th grade level. The consent forms will be translated into easy-to-read Thai language versions, and the translation will be verified by blinded back translation. The enrollment consent form will be reviewed with the Chiang Rai Microbicide Research Community Advisory Group (see Introduction). A dedicated study research nurse will be present and will explain the informed consent form to each woman at the time of enrollment. The protocol must be approved by all Institutional Review Boards (the Population Council IRB, the U.S. CDC IRB, and the Thai Ministry of Public Health Ethical Review Committee) before the first study participant is screened.

Women will be informed that they are completely free to refuse participation or to withdraw from the study anytime after enrollment, without consequence to their medical care. Investigators will provide confidentiality for participants, to the extent allowable by law, and their names will not appear on study forms. All laboratory specimens, evaluation forms and records will be identified only by a coded number to maintain participant confidentiality. All records will be kept in a locked filing cabinet at each study site. Data will be electronically transmitted only in a form that cannot be identified with the participant. Only the study staff will have access to a separate file where participant names are linked to identification numbers. Clinical information will not be released without the permission of the participant, except as required for monitoring by the study sponsor (The Population Council). National and international regulatory agencies may also request access to records, including medical record, but the identity of individual participants will remain confidential in such instances.

Each potential study participant will sign an informed consent for screening (Appendix 1) prior to the screening on Day -30. Signed informed consent will also be required from study participant husbands/steady partners (Appendix 2) at the first baseline visit (enrollment). Women who are eligible and who choose to participate in the study will sign an informed consent for enrollment (Appendix 3) at the first baseline visit (enrollment). The informed consent forms will be filed in a locked filing cabinet at the study clinic or the TUC Chiang Rai office with the Study Participant Identification Register. Keys will be available only to study staff.

As part of the Population Council’s monitoring responsibilities, the participant will be asked to acknowledge that an interview may be requested by a representative of the Population Council to determine whether informed consent was given. If an interview is requested, the participant will have the option of accepting or declining the interview.

All participants will be given the name and telephone number of the local study coordinator for any questions about the study or problems during the study, including if she feels that she is experiencing side effects or complications of the study products. All participants will be given the name and telephone number of an independent source of information (a nurse in the clinic not associated with the study) if there are questions about the way in which the study is being conducted. All participants will be given the name and telephone number of a Ministry of Public Health Ethical Review Committee official for use if there are questions about the rights of a study subject.

# Adverse events

Any untoward medical occurrence in a participant during the trial should be reported as an adverse event (AE), whether or not there is a relationship with the study product. Investigators should rate the adverse event as Aunrelated,” “possibly related,” or Aprobably related” to the study product, as shown on the adverse event forms. Therefore, all reproductive tract infections, (except HPV), including asymptomatic infections and HIV infection, all abnormal laboratory results, any abnormality detected during the gynecological/visual examination, and discomfort (including painful sexual intercourse and painful urination), should be considered an adverse event. A recurrence of signs, symptoms or infections reported as adverse events on previous forms, but for which adequate treatment was given, should be considered as new adverse events.

# Serious adverse events

A serious adverse event (SAE) or reaction which requires expedited reporting is defined by the U. S. Food and Drug Administration (FDA) as any untoward medical occurrence that:

- results in death or is life-threatening; (“Life-threatening” is defined as a SAE in which the patient was at risk of death at the time of the event. It does not refer to an event which hypothetically might have caused death if it were more severe);
- requires hospitalization or prolongs existing hospitalization;
- results in persistent or significant disability/incapacity;
- is a congenital abnormality/birth defect;
- jeopardizes participant health and required medical/surgical intervention to prevent serious outcome; or
- any other event that the investigator considered serious. (The Population Council also considers cancer and drug overdose as serious adverse events.)

Medical and scientific judgment should be exercised in deciding whether expedited reporting is appropriate in other situations, such as important medical events that may not be immediately life-threatening or result in death or hospitalization but may jeopardize the patient or may require intervention to prevent one of the other outcomes listed in the definition above. Generally, these should also be considered serious. Examples of such events are intensive treatment in an emergency room or at home for allergic bronchospasm; blood dyscrasias or convulsions that do not result in hospitalization; or development of drug dependency or drug use.

A probable relationship with the study product is *not* necessary to trigger reporting a SAE (i.e. car accidents still have to be reported if they reach the attention of the investigator).

All SAEs, whether or not related to study product use, must be reported by the investigator within 24 hours of his/her becoming aware of them to the Population Council SAE Desk (212-327-8673), to the Population Council study coordinator, Sarah Braunstein (212-339-0638), and to the CDC IRB. This reporting can be done by telephone, fax or email. The report of SAE by phone or fax must always be followed by a detailed written report, due within 7 days of the initial report. The Population Council study coordinator will inform the Population Council IRB. The local principal investigators will inform the U.S. CDC IRB and provide a Thai-language report to the Thai Ethical Review Committee.

SAE that are unexpected, fatal, or life-threatening and related to gel use are to be reported by the Population Council to the U.S. FDA, and the Population Council IRB, as soon as possible, but no later than 7 calendar days after first knowledge by the investigators that a case qualifies, followed by as complete a report as possible within 8 additional calendar days. This report must include an assessment of the importance and implications of the findings, including relevant previous experience with the same or similar products.

SAEs that are not fatal or life-threatening but are unexpected and possibly related to gel use must be filed by the Population Council with the US FDA as soon as possible, but no later than 15 calendar days after first knowledge that the case meets the minimum criteria for expedited reporting.

SAEs that meet only one of the criteria B unexpected or related to gel use B are to be reported in the annual report to the regulatory bodies.

Information for final description and evaluation of a SAE case report may not be available within the required time frame for reporting outlined above. Nevertheless, for regulatory purposes, initial reports should be submitted to the US FDA and the Thai FDA within the prescribed time as long as the following minimum criteria are met: an identifiable patient; a suspect medicinal product; an identifiable reporting source; and an event or outcome that can be identified as serious and unexpected, and for which there is a reasonable suspected causal relationship to the study product. Follow-up information should be actively sought and submitted as it becomes available.

As required by regulatory authorities, all serious and non-serious adverse reactions to the study product will be included as part of the semi-annual or annual progress reports.

When reporting a serious adverse event to the Population Council study coordinator, the investigator should protect the patient’s confidentiality by excluding names and addresses. The study participant’s unique identification number and enrollment date should be used in the report and the investigator should retain the code to facilitate verification of data by the Population Council study coordinator or drug regulatory authority. The name of the investigator reporting the SAE should be stated. In all cases of SAEs where a relationship with the study product is probably related, study product use should be discontinued immediately. Appropriate treatment should be given.

# Study monitoring

The Population Council is responsible for ensuring protocol adherence and validity of data recorded. The Population Council has designated monitors for this study. The Population Council study monitor located in Bangkok will visit the study site regularly to monitor the study. A Population Council study monitor based in New York will conduct two site visits during the study. The first site visit will be 2-3 months after enrollment has begun, and the second will be at the end of the study. Study participants will be informed that an interview may be requested by a representative of the Population Council during the study and that if an interview is requested, the study participant has the option of accepting/declining the interview. Study monitors will be given access to primary source documentation to confirm data collection and recording accuracy. The monitors’ duties are to aid investigators in maintaining complete, legible, well-organized, and easily retrievable data. In addition, the monitors will explain, interpret and ensure the investigators’ understanding of the protocol, reporting responsibilities, and data validity.

# Product interruption criteria

Application of the study product will be interrupted in the following circumstances:

- Pregnancy
- Genital or other major surgery
- Medical condition incompatible with the study protocol or visit schedule
- SAE related to the investigational product
- Consent withdrawn
- Missed follow-up visit. However, at the investigators’ discretion, a participant may resume study participation (after her next menstrual period if menstruating)
- Lack of compliance

If an emergency, such as anaphylactic shock occurs, arises and it is necessary to unblind the product used by a particular participant, four individuals will have access to the randomization scheme, and can be reached both at work and at home, 24 hours a day, at the following telephone numbers:

Barbara Friedland (NY): work: 212-339-0629 home: 212-865-0657

Beverly Winikoff (NY): work: 212-339-0623 home: 212-685-7806

Robin Maguire (NY): work: 212-327-8729 home: 914-944-4135

Philip Guest (Bangkok): work: 02-251-4766 home: 02-255-5759 mobile: 01-848-3625

# Trial closure considerations

The Population Council reserves the right to terminate the study prematurely if study participant recruitment is too slow, if study participant retention in the study is insufficient, if undue risk related to the study intervention arises, or if there is any deviation from the trial protocol. Related expanded safety and acceptability studies of Carraguard are underway in Chiang Rai and in South Africa. Results from those studies are scheduled to become available while this clinical trial is underway. Data from those studies or other research when available may be used to help decide whether to modify or terminate this clinical trial if there is evidence of undue risk related to the study intervention.

# Protocol amendments

Changes to the protocol will be made only when written protocol amendments, signed by the principal investigators and the project director, have been approved by the local, Population Council, and US CDC IRBs. After IRB approval of an amendment, a copy of the amendment signed by each of the Principal Investigators will be kept on file at the Population Council in New York and at CDC and TUC. If the protocol change does not increase the risk of harm to the participants, the chairs of the IRBs may approve such changes in advance of the full committee meeting. Protocol amendments may affect consent forms for current and future patients, which will have to be modified accordingly, and also approved by the ethical committees.

Reference List

(1) HIV/AIDS: the global epidemic. World Health Forum 1997; 18(3-4):369-372.

(2) Kilmarx PH, Supawitkul S, Wankrairoj M, Uthaivoravit W, Limpakarnjanarat K, Saisorn S et al. Explosive spread and effective control of human immunodeficiency virus in northernmost Thailand: the epidemic in Chiang Rai province, 1988-99. AIDS 2000; 14(17):2731-2740.

(3) Information on File at the Population Council and with the FDA (IND 58-441). 2001.

(4) United States Food and Drug Administration. GRAS (Generally Recognized as Safe) Food Ingredients -- Carrageenan, report by Informatics, Inc. for the U.S. Food and Drug Administration. 2001. Springfield, VA, National Technical Information Service. 1972.

(5) Elias CJ, Coggins C, Alvarez F, Brache V, Fraser IS, Lacarra M et al. Colposcopic evaluation of a vaginal gel formulation of iota-carrageenan. Contraception 1997; 56(6):387-389.

(6) Coggins C, Blanchard K, Alvarez F, Brache V, Weisberg E, Kilmarx PH et al. Preliminary safety and acceptability of a carrageenan gel for possible use as a vaginal microbicide. Sex Transm Infect 2000; 76(6):480-483.

(7) Mostad SB, Overbaugh J, DeVange DM, Welch MJ, Chohan B, Mandaliya K et al. Hormonal contraception, vitamin A deficiency, and other risk factors for shedding of HIV-1 infected cells from the cervix and vagina [see comments]. Lancet 1997; 350(9082):922-927.

(8) Goulston C, McFarland W, Katzenstein D. Human immunodeficiency virus type 1 RNA shedding in the female genital tract. Journal of Infectious Diseases 1998; 177(4):1100-1103.

(9) John, GC, Nduati RW, Mbori-Ngacha D, Overback J, Welch M, Richardson Ba et. Al. Genital shedding of human immunodeficiency virus type 1 DNA during pregnancy: association with immunosuppression, abnormal cervical or vaginal discharge, and severe vitamin A deficiency. Journal of Infectious Diseases 1997; 175(1):57-62.

(10) Ghys PD, Belec L, Diallo MO, Ettiegne-Traore V, Becquart P, Maurice C et al. Cervico-vaginal anti-HIV antibodies in HIV-seronegative female sex workers in Abidjan, Cote d'Ivoire. AIDS 2000; 14(16):2603-2608.

(11) Cohen MS, Hoffman IF, Royce RA, Kazembe P, Dyer JR, Daly CC et al. Reduction of concentration of HIV-1 in semen after treatment of urethritis: implications for prevention of sexual transmission of HIV-1. AIDSCAP Malawi Research Group. Lancet 1997; 349(9069):1868-1873.

(12) Mostad SB, Jackson S, Overbaugh J, Reilly M, Chohan B, Mandaliya K et al. Cervical and vaginal shedding of human immunodeficiency virus type 1-infected cells throughout the menstrual cycle. Journal of Infectious Diseases 1998; 178(4):983-991.

(13) Chuachoowong R, Shaffer N, Siriwasin W, Chaisilwattana P, Young NL, Mock PA et al. Short-course antenatal zidovudine reduces both cervicovaginal human immunodeficiency virus type 1 RNA levels and risk of perinatal transmission. Bangkok Collaborative Perinatal HIV Transmission Study Group. Journal of Infectious Diseases 2000; 181(1):99-106.

(14) Panther LA, Tucker L, Xu C, Tuomala RE, Mullins JI, Anderson DJ. Genital tract human immunodeficiency virus type 1 (HIV-1) shedding and inflammation and HIV-1 env diversity in perinatal HIV-1 transmission. Journal of Infectious Diseases 2000; 181(2):555-563.

1. Mauck C, Rosenberg Z, Van Dumme L, for the International Working Group on Microbicides. Recommendations for the clinical development of topical microbicides: an update. 15, 857-868. 5-4-2001.
2. CONRAD and World Health Organization. Manual for the standardization of colposcopy for the evaluation of vaginal products. 2000. Geneva, Switzerland.

(17) Young N, Kilmarx P, Borchardt K, Chaowanachan T, Wasinrapee P, Suksripanich O. Inhibitory effects of vaginal microbicides on COBS Amplicor and Gen-Probe Chlamydia trachomatis and Neisseria gonorrhoeae, and InPouch TH Trichomonas vaginalis tests [abstract]. 1. Berlin, International Congress of Sexually Transmitted Infections.

# Appendices

Appendix 1: Screening visit informed consent (women)

Appendix 2: HIV testing and partner’s study participation informed consent (men)

Appendix 3: Enrollment informed consent (women)

Appendix 4: Table 1. Study visits, based upon ~28 day menstrual cycle

Appendix 5: Table 2. Schedule of clinic visits and data collection

Appendix 6: Table 3. Forms schedule

Appendix 7: Table 4. Laboratory tests, specimens, and processing laboratories

# Study forms

Eligibility checklist

CRH referral letter

CRH Assessment form

Clinician screening form

Male HIV test form

Comprehension test

Enrollment form

Enrollment interview

Clinician study form

Interview follow-up form

Acceptability questionnaire

Closing form

Master checklist

Product distribution log

Applicator usage log

Study gel interruption form

Adverse events form

Severe adverse events form

Concomitant medications log

**Appendix 1**

**A RANDOMIZED, CONTROLLED, DOUBLE-BLIND, CROSS-OVER TRIAL OF SAFETY, EFFECT ON GENITAL TRACT HIV SHEDDING, AND ACCEPTABILITY OF VAGINAL USE OF CARRAGUARD BY**

**HIV-INFECTED WOMEN**

**INFORMED CONSENT (women) for SCREENING VISITS #1, #2, and HIV Evaluation**

**CHIANG RAI, THAILAND**

**(Completed at screening visit #1)**

[Flesch-Kincaid reading level 8.3. Will be translated into easy-to-read Thai and verified by back translation]

The Thailand Ministry of Health and US CDC Collaboration and the Population Council are doing this research study. The Thailand Ministry of Health and US CDC Collaboration is a joint activity of the Thai Ministry of Health and the U.S. Centers for Disease Control and Prevention. Women will be screened until 54 have enrolled in the study. Women who are eligible for enrollment will have three clinic visits each month for the three months they are enrolled in the study.

Study purpose

There are three main reasons for this study:

1. To see if Carraguard vaginal microbicide gel is safe for use by HIV-infected women. We would like to see if it causes any problems in the vagina, such as burning, itching, pain or soreness.
2. To see if Carraguard changes the amount of HIV in the vagina.

3. To find out what women think about using this microbicide gel.

Study Products

1) Carraguard: The microbicide gel being tested for safety is called Carraguard. It is made from carrageenan, which comes from seaweed. Carrageenan is used in many foods, like ice cream and puddings. It is also used in make-up, creams and lotions.

-In lab tests, Carraguard microbicide gel has been shown to block HIV - the virus that causes AIDS, and other sexually transmitted infections (or STIs).

-Scientists hope that some day Carraguard might protect women from getting HIV and other STIs.

- Carraguard has not yet been shown to prevent infection in humans. Scientists first want to make sure it is not harmful.

-So far, 35 women in 5 countries used a microbicide gel very similar to Carraguard once a day for a week. It did not cause harm. Studies are now going on in Chiang Rai with 165 women and in South Africa with 400 women using Carraguard or a placebo gel. Those studies aren’t finished yet so we don’t know the results. *(We will update this information once we have the results of these studies).*

2) Placebo: The placebo gel is made from methyl cellulose, which is known to be safe. It is found in many products, like eye drops, pain medicine, and skin rash medicine. It has also been used before in studies like this.

-Neither of the gels will stop you from getting pregnant. You should use a family planning method while in the study.

-It is not known if the gels prevent HIV or other STIs.

-The gels also cannot cure AIDS or STIs.

-Your partner need to use condoms while you are in this study to prevent transmission of different kinds of HIV between partners, and we will provide you with condoms.

Information and testing process

The purpose of this clinic visit is to tell you about the study, to offer you HIV testing and counseling, and to ask you to sign a consent, because you may be exposed to the study gels.

This clinic visit will have two parts:

1. Information session: This will include describing the study, including risks and benefits to you, and answering any questions you have.
2. HIV pre-test counseling and testing: Someone from the study team will speak with you about HIV infection, safer sex, and how to use condoms. About two teaspoons of blood will be taken from your arm to test for HIV, the virus that causes AIDS. We will ask you to return to the clinic after two weeks to get your test results.

At any point in this process you may decide not to participate.

Study steps

-Women will be enrolled in this study for three months. During two of the months, women will be asked to insert a gel ( Carraguard or a gel like Carraguard, called a Aplacebo”) into their vagina once a day for one week. During the other month, women will insert no gel into their vagina.

Screening process purpose

The purpose of the screening is to see if you can take part in this study. The screening involves two screening visits to this clinic and one visit to the Chiang Rai Hospital for an HIV care evaluation. At the first screening visit, you will be asked a set of questions and will have your blood drawn for an HIV test.

You will then be asked to visit a doctor at Chiang Rai Hospital who will talk to you about your HIV infection and other problems, such as tuberculosis, that you may experience as a result of your HIV infection. If you test positive for tuberculosis, you will be offered 9 months of preventive therapy. You will not need to pay for this clinic visit. This visit and other medical expenses related to your HIV infection up to 1000 baht will be paid for by the study. If you are part of the government health program in which you pay 30 baht for every medical visit which covers the visit and any care they receive, this money will be given directly to the hospital by a study nurse at the time of your visit for the care you receive; in some cases (like if you pay for your healthcare in another way), the money will be given to you by the study staff if you bring your receipts for medical care you paid for to the clinic. Expenses for HIV related treatment beyond the 1000 baht will have to be paid by you. Medicines (and the cost for any of these medicines) for treating HIV infection will not be provided as a part of this study.”

You will then come to this clinic for a the second screening visit when you will be asked another set of questions by the study staff and will have a full examination, including a pelvic examination, with lab testing. You will be asked to return to the clinic after two weeks to get your test results.

If you have a husband or steady partner with whom you will be sexually active during the study, we will ask you to have him come to the clinic to have an HIV test. Because he may have contact with th one of the study gels being tested for safety, he will need to give his informed consent prior to your participation in the study.

You can enroll in the study if you are eligible, you choose to do so, and your partner tests HIV positive and gives his consent. Even if the study staff find that you are eligible for the study, you can still decide not to be in the study.

*1) Interview and Counseling*

A member of the study staff will ask you questions at both of the screening clinic visits. She will ask your age, if you have children, if you are married, what kind of family planning method you use, and if you use any vaginal products. She will also ask questions about your sexual practices, including if you use condoms or not. All of the answers you give will be kept private whether or not you join the study. Someone from the study team will talk with you about safer sex and about how to use condoms.

*2) Clinical Exams*:

At the first screening visit, the study nurse will:

-Take about two teaspoons of blood from your armto test for HIV, the virus that causes AIDS, and to test for syphilis.

-Take a sample of your urine to test for pregnancy.

-Using a speculum, a special instrument to help check inside your vagina, do a pap smear for signs of cervical cancer.

At the first screening visit, the study staff will schedule you for another clinic visit in 1 week to see a doctor who can help you take care of your HIV infection. One of the study staff will take you to the Chiang Rai Hospital Department of Medicine for this evaluation.

At the second screening visit, the study nurse will check your general health, including a complete gynecological exam. At this exam, she will:

-Use a speculum, a special instrument to help check inside your vagina.

-Look inside your vagina to see if you have any ulcers or sores.

-Take swabs from your vagina and cervix. The swabs will be checked at a lab to see if you have any infections.

-Photos of areas of your vagina or cervix may be taken at this time. The photos may be used in reports, but not with your name.

HIV testing

To participate in this study, your steady sex partner/husband must also be HIV positive. Before you are enrolled for this study, your partner must come to the clinic to be tested for HIV infection. The study staff will inform him that he has HIV infection, about the purpose, risks, and benefits of the study, and he will have the opportunity to ask questions. He will not be given any information about your medical record or answers that you may have given during interviews with the study staff. Women who have a steady partner/husband who she will not see during the study period or with whom she is not sexually active will not be required to bring their partner to the clinic for HIV testing and informed consent.

Sexually transmitted infection testing:

If your test results show that you have an infection that can be cured, the study nurse will give you medicine or you might be called back before your next clinic visit(You must be treated for this infection before you can be enrolled in the study).If you agree, your partner will also be told about the infection and given treatment.

If you have genital ulcer disease, you will be treated for curable infections (syphilis and chancroid) right away, and a test will be conducted after the study is completed to identify which infection caused your ulcer.

□ Yes, I would like to be contacted after the end of the study with the results of any genital ulcer disease testing.

□ No, I do not want to be contacted after the end of the study with the results of genital ulcer disease testing.

You will have a pap smear as part of the study screening process, and you will be informed of these results. You will have tests for a virus that may cause abnormal pap smears and may lead tocervical cancer. You will not be given these results. It is important that you have regular annual pap smears which is the best way to check for cervical cancer.

Burdens and Risks

Possible burdens and risks from screening are:

-You may feel embarrassed by some of the questions you are asked.

-You could have soreness or bruising from the needle when blood is taken from your arm for testing.

-You could feel uncomfortable when the nurse checks inside your vagina. There could be a small amount of bleeding afterward.

-You may feel upset if you find out you have an STI.

-If you find out you have an STI, it could cause problems in your relationships and/or family.

Benefits

The main thing that you would gain from this screening process would be the safe-sex counseling and the limited medical check-up, and care already listed. By getting screened to be in the study, you can find out if you have an STI which you might not have known about. You will be able to get treated. If you wish, your partner will be referred and given treatment for the infection at no cost.

Reimbursement

You will receive 300 baht (at the screening visit #2) for your time and transportation costs for taking part in this screening process. Your husband/partner will be reimbursed (50 baht transport fee) for his transportation costs for coming to the clinic to have an HIV test and learning about the study.

Alternative ways to get testing and treatment if you do not want to be in this study

You can get some, but not all, of the counseling, testing, and treatment like that being offered in this study at Chiang Rai Hospital and at other clinics in this area, even if you are not part of this study. However, you will have to pay for these services.

Confidentiality

If you have a husband or steady partner with whom you will be sexually active during the study, he must be told (by you or the study staff) that you have HIV infection and about your taking part in this study. Your answers to the interview questions, your exam results, and your other test results will not be shared with other people, such as your husband or your doctor, without your permission, unless required by law. Most study documents will only have a code number and will not have your name. All study documents will be kept in locked files. We will send reminder letters for your follow-up appointments. If you miss an appointment we will try to contact you by sending letters, telephoning, and/or visiting you. We would also like the name of person we may contact if we cannot contact you. We will not put the reason for the visit in the reminder letters or tell it to others.

Voluntary participation

Taking part in this study screening process is up to you. You may stop taking part at any time. You may refuse to answer any of the questions. Your medical care will not be affected if you decide not to take part in the study screening process or if you decide to stop taking part at a later date.

We may ask you to have an interview with a representative of the Population Council during the study so we can understand whether the study is being done well. If someone asks you to talk about the study, you can say “yes” or “no.” If you say “yes,” all facts from the meeting will be kept private.”

At any time during the study, if you have questions, concerns, or unusual symptoms, please return to the clinic promptly to meet with a study nurse or to have a clinical exam.

If you have questions about the study screening process or if you believe you have been injured as a result of being in the study screening process

You can call Khun Supaporn Chaikummao at the Chiang Rai Health Club (tel. 711-234) or (tel: 01-8504343 cell, 24 hours). Or you can call Dr. Somsak Supawitkul, Deputy, Office of Preventive Health, Chiang Rai Public Health Office (tel. 711-911, 711-403 ).

If you have questions about whether you should agree to be in the study screening process or about the way the study screening process is being done

You can talk to Wanida Maneerut, Nurse, Chiang Rai Provincial Public Health Office Tel 711-911 ext. 110.

If you have questions about your rights as a study subject

You can call the Secretary to Ethical Review Committee, at the Ministry of Public Health in Nonthaburi (tel. 02-591-8249; fax. 02-591- 8245).

Participant’s statement

I have had a chance to ask questions about the study screening process. My questions have been answered.

I have had a chance to ask questions and my questions have been answered to my satisfaction about the purpose of the study screening process, the procedures, and the risks and benefits.

I agree to take part in this study screening process, to follow the procedure, and to come to all study visits as best as I am able.

I have had a chance to ask questions and my questions have been answered to my satisfaction about that the fact that I may stop taking part in this study screening process at any time without affecting my right to receive regular medical care.

Name of Participant: ________________________________

_______________ _________________________________ ____________

Date Signature of Participant * (thumbprint)

*In case the woman is not able to sign this form, this attests that the consent form has been read and explained accurately by a member of the research staff, and that the woman has fixed her thumbprint as consent.

We would like to keep any blood or other fluids that are left over after we do your tests. We may use these samples for future tests such as other infections; however, we will not do any human genetic testing on your specimen. We will keep these samples with some facts about you such as your age and sex. We will not put your name on the samples so we would not be able to report the results of any future tests back to you.

□ Yes, I have had a chance to ask questions and my questions have been answered to my satisfaction about the fact that any blood or other fluids that are left over after my tests are done will be kept.

□ No, I do not want any blood or other fluids that are left over after my tests are done to be kept.

Name of Participant: ________________________________

_______________ _________________________________ ____________

Date Signature of Participant * (thumbprint)

*In case the woman is not able to sign this form, this attests that the consent form has been read and explained accurately by a member of the research staff, and that the woman has fixed her thumbprint as consent.

Study Team Member’s Statement

I, the undersigned, have explained to the volunteer in a language that she understands, the procedures to be followed in the study screening process, the risks and benefits involved, and the obligations of the study team.

_______________ _________________________________

Date Signature of Study Team Member

_______________ _________________________________

Date Signature of Witness to the Above

Signatures and Explanations

**Appendix 2**

**A RANDOMIZED, CONTROLLED, DOUBLE-BLIND, CROSS-OVER TRIAL OF SAFETY, EFFECT ON GENITAL TRACT HIV SHEDDING, AND ACCEPTABILITY OF VAGINAL USE OF CARRAGUARD(BY**

**HIV-INFECTED WOMEN**

**INFORMED CONSENT (men) B for HIV TESTING AND PARTNER STUDY PARTICIPATION**

**CHIANG RAI, THAILAND**

**(To be completed prior to participant’s enrollment)**

[Flesch-Kincaid reading level 8.5. Will be translated into easy-to-read Thai and verified by back translation.]

Your partner is interested in participating in a research study conducted by the Population Council from Bangkok and the U.S. and the Thailand Ministry of Health and US CDC Collaboration (TUC). The TUC a joint activity of the Thai Ministry of Health and the U.S. Centers for Disease Control and Prevention. The study in which your partner is participating, along with risks and benefits to you, is described below.

Purpose of today’s visit

For your wife/partner to participate in this study, you must agree to be tested for HIV infection, you must have a positive HIV test, and you need to sign an informed consent form stating that you agree to be tested for HIV and that you may be exposed to a microbicide gel being tested for safety. Your partner may not participate in the study unless you sign the consent form.

Study purpose

There are three main reasons for this study:

1. To see if Carraguard vaginal microbicide gel is safe for use by HIV-infected women. We would like to see if it causes any problems in the vagina, such as burning, itching, pain or soreness.
2. To see if Carraguard changes the amount of HIV in the vagina.
3. To find out what women’s experiences are using this microbicide gel.

Study Products

1) Carraguard: The microbicide gel being tested for safety is called Carraguard. It is made from carrageenan, which comes from seaweed. Carrageenan is used in many foods, like ice cream and puddings. It is also used in make-up, creams and lotions.

-In lab tests, Carraguard microbicide gel has been shown to block HIV - the virus that causes AIDS, and other sexually transmitted infections (or STIs).

-Scientists hope that some day Carraguard might protect women from getting HIV and other STIs.

- Carraguard has not yet been shown to prevent infection in humans. Scientists first want to make sure it is not harmful.

-So far, 35 women in 5 countries used a microbicide gel very similar to Carraguard once a day for a week. It did not cause harm. Studies are now going on in Chiang Rai with 165 women and in South Africa with 400 women using Carraguard or a placebo gel. Those studies aren’t finished yet so we don’t know the results. *(We will update this information once we have the results of these studies).*

2) Placebo: The placebo gel is made from methyl cellulose, which is known to be safe. It is found in many products, like eye drops, pain medicine, and skin rash medicine. It has also been used before in studies like this as a placebo.

-Neither of the gels will stop your wife/partner from getting pregnant. She should use a family planning method while in the study.

-It is not known if the gels prevent HIV or other STIs.

-The gels also cannot cure AIDS or STIs.

-You need to use condoms while being in this study to prevent transmission of different kinds of HIV between partners, and we will provide you with condoms.

Information and testing process

The purpose of this clinic visit is to tell you about the study, to offer you HIV testing and counseling, and to ask you to sign a consent, because you may be exposed to the study gels.

This clinic visit will have two parts:

1)Information session: This will include describing the study, including risks and benefits to you, and answering any questions you have.

2)HIV pre-test counseling and testing: Someone from the study team will speak with you about HIV infection, safer sex, and how to use condoms. About two teaspoons of blood will be taken from your arm to test for HIV, the virus that causes AIDS. We will ask you to return to the clinic after two weeks to get your test results.

At any point in this process you may decide not to participate.

Study steps

-Women will be enrolled in this study for three months. During two of the months, women will be asked to insert a gel ( Carraguard or a gel like Carraguard, called a Aplacebo”) into their vagina once a day for one week. During the other month, women will insert no gel into their vagina.

HIV/STI testing

If you do have HIV, the study staff will offer you counseling and help you to find care available in the area. The study staff will also provide testing and treatment for sexually transmitted infections (STIs) to your partner at no charge to you. Medicines for treating HIV infection will not be provided to you or your partner as a part of this study.

If any of the other tests show that your partner has a curable sexually transmitted disease, she may refer you to the clinic to get medicine for it.

Burdens and Risks

Possible burdens and risks for you of having HIV testing:

-You could have soreness or bruising from the needle when blood is taken from your arm for testing.

-You may feel upset if you find out you have an STI or HIV.

Possible risks for you of your partner participating in this study are:

-You or your partner might have burning, itching, pain, or soreness using either study gel. -Based on what is known about both gels, this is not very likely. Using the Carraguard microbicide gel could increase your risk of getting certain STIs. This is also not very likely.

Benefits

The main thing that you would gain from participating in this process would be HIV testing, safe-sex counseling, and referral for HIV care.

Alternative ways to get testing and treatment if you do not want to be in this study

You can get some of the HIV counseling, testing, and treatment for STIs like that being offered in this study at Chiang Rai Hospital and at other clinics in this area, even if you are not part of this study. However, you will have to pay for these services.

Reimbursement

You will be given 50 baht cash at your clinic visit for your transportation costs for this visit.

Confidentiality

Your answers and your HIV test results will not be shared with anyone else without your permission, unless required by law. Your partner/wife will not be given your study results but she may understand that if she is allowed to participate in the study, you must be HIV-infected. Most study documents will only have a code number and will not have your name. All study documents will be kept in locked files. If you miss the HIV results appointment, we will try to contact you by sending letters, telephoning, and/or visiting you. With your permission, we may contact a person named by you to try to contact you. We will not put the reason for the visit in the reminder letters or tell it to others.

Voluntary Participation

Taking part in this study testing process is up to you. You can refuse to participate without affecting your right to receive regular medical care.

At any time during the study, if you have questions, concerns, or unusual symptoms, please return to the clinic promptly to meet with a study nurse or to have a clinical exam.

If you have questions about the study screening process or if you believe you have been injured as a result of being in the study testing process

You can call Khun Supaporn Chaikummao at the Chiang Rai Health Club (tel. 711-234) or (tel: 01-8504343 cell, 24 hours) . Or you can call Dr. Somsak Supawitkul, Deputy, Office of Preventive Health, Chiang Rai Public Health Office (tel. 711-911, 711-403 ).

If you have questions about whether you should agree to be in the study testing process or about the way the study testing process is being done

You can talk to Wanida Maneerut, Nurse, Chiang Rai Provincial Public Health Office Tel 711-911 ext. 110.

If you have questions about your rights as the partner of a study subject

You can call the Secretary to Ethical Review Committee, the Ministry of Public Health in Nonthaburi (tel. 02-591-8249; fax. 02-591- 8245).

Participant’s statement

-I have had a chance to ask questions about the study and the HIV testing process. -My questions have been answered.

-I have had a chance to ask questions and my questions have been answered to my satisfaction about the purpose of the HIV testing process, the procedures, and the risks and benefits to me of being HIV tested today and of my partner participating in this study.

-I agree to take part in this HIV testing process.

-I agree to have my partner participate in this study.

-I have had a chance to ask questions and my questions have been answered to my satisfaction about the fact that I may refuse to participate at any time without affecting my right to receive regular medical care.

I give the study staff permission to contact me with the contact information I have

provided if necessary. Yes [ ]

No [ ]

Name of Participant: ________________________________

_______________ _________________________________ ____________

Date Signature of Participant * (thumbprint)

*In case the man is not able to sign this form, this attests that the consent form has been read and explained accurately by a member of the research staff, and that the man has fixed his thumbprint as consent.

Study Team Member’s Statement

I, the undersigned, have explained to the volunteer in a language that he understands, the procedures to be followed in the HIV testing process and in his partner’s participation in the study, the risks and benefits involved for him, and the obligations of the study team.

_______________ _________________________________

Date Signature of Study Team Member

_______________ _________________________________

Date Signature of Witness to the Above

Signatures and Explanations

**Appendix 3**

**A RANDOMIZED, CONTROLLED, DOUBLE-BLIND, CROSS-OVER TRIAL OF SAFETY, EFFECT ON GENITAL TRACT HIV SHEDDING, AND ACCEPTABILITY OF VAGINAL USE OF CARRAGUARD() BY**

**HIV-INFECTED WOMEN**

**INFORMED CONSENT B ENROLLMENT (women)**

**CHIANG RAI, THAILAND**

[Flesch-Kincaid reading level 8.5. Will be translated into easy-to-read Thai and verified by back translation]

The Population Council from Bangkok and the U.S. and the Thailand Ministry of Health and US CDC Collaboration (TUC) are doing this research study. The TUC is a joint activity of the Thai Ministry of Health and the U.S. Centers for Disease Control and Prevention.

Study purpose

There are three main reasons for this study:

1. To see if Carraguard vaginal microbicide gel, a gel made from seaweed which scientists hope one day might protect women from HIV infection, is safe for use by HIV-infected women. We would like to see if it causes any problems in the vagina, such as burning, itching, pain, or soreness.
2. To see if Carraguard changes the amount of HIV in the vagina.
3. To find out about how women feel about using this microbicide gel.

Study Products

1) Carraguard: The microbicide gel being tested for safety is called Carraguard. It is made from carrageenan, which comes from seaweed. Carrageenan is used in many foods, like ice cream and puddings. It is also used in make-up, creams and lotions.

-In lab tests, Carraguard microbicide gel has been shown to block HIV - the virus that causes AIDS, and some other sexually transmitted infections (or STIs).

-Scientists hope that some day Carraguard might protect women from getting HIV and other STIs.

- Carraguard has not yet been shown to prevent infection in humans. Scientists first want to make sure it is not harmful.

-So far, 35 women in 5 countries used a microbicide gel very similar to Carraguard once a day for a week. It did not cause harm. Studies are now going on in Chiang Rai with 165 women and in South Africa with 400 women using Carraguard or a placebo gel to see if the microbicide gel is safe for HIV negative women. Those studies aren’t finished yet so we don’t know the results. *(We will update this once we have the results of these studies).*

2) Placebo: The placebo gel is made from methyl cellulose, a lubricant which is known to be safe. It is found in many products, like eye drops, pain medicine, and skin rash medicine. It has also been used before in studies like this as a placebo.

-Neither of the gels will stop you from getting pregnant. You should use a family planning method while in the study. You cannot be in the study if you are pregnant or trying to get pregnant within the next 3-4 months. Please inform the study staff if you suspect you are pregnant.

-It is not known if the gels prevent HIV or other STIs.

-The gels also cannot cure AIDS or STIs.

-You need to use condoms while in this study, and we will provide you with condoms.

Steps to be followed

- During the three month study, you will have three clinic visits each month- the baseline, the first follow-up, and the second follow-up visit. You will have a full exam by a study nurse three times a month for the three months with one final visit, for a total of 10 visits.

- You will use each of the two products ( Carraguard or a gel like Carraguard, called a Aplacebo”) during two months and will have one month in which you use no product.

-

- For one week during the two months when you are using a gel, you will be asked to either put the Carraguard microbicide gel or the placebo gel in their vagina once a day. Neither you nor the staff at the study site will know which of the two gels you are using.

- At each visit, you will have a pelvic exam using a speculum, and the nurse will check inside your vagina. She will look for problems that might be due to the microbicide gel.

We would like to tell you more about each type of study visit:

Baseline visits:

**-**During the first clinic visit each month, the baseline visit, you will have the following tests: a urine pregnancy test, a blood test for the amount of HIV, a pelvic exam when the study nurse will take 4 swabs from your vagina and cervix to check for infections, and a test to measure the amount of HIV in your vagina, and a test for semen which might affect the amount of HIV in the vagina. To measure the amount of HIV in your vagina, during each pelvic exam, the nurse will collect samples using two swabs of your vagina and cervix and will rinse your vagina with a tablespoon of sterile salt water to collect fluid for testing. At the baseline visit, you will be examined two times. The second exam will be about 15 minutes after the first exam. At that second exam, the nurse will use four swabs and will rinse your vagina and one time only or two times if needed to get a specimen to measure the amount of HIV in your vagina again..

-At two of the baseline visits, you will be asked to put the study gel into your vagina before your second pelvic exam.

-At the three baseline visits and the final visit, the study nurse will use an instrument to get a better look during your pelvic exam (A colposcope is a magnifying light. It does not touch your body).

-Photos of areas of your vagina or cervix may be taken at this time. The photos may be used in reports, but not with your name.

Follow-up visits:

- You will be asked to come to the clinic two more times during the month, at the first follow-up visit and the second follow-up visit.

- At each of those two follow-up visits, the study nurse will take 4 swabs from your vagina and cervix to check for infections, and test to measure the amount of HIV in your vagina (as described above).

- At the first follow-up visit, the study nurse will also use the instrument (described above) to get a better look during your pelvic exam. Photos of areas of your vagina or cervix may be taken at this time. The photos may be used in reports, but not with your name.

At any time during the study, if you have questions, concerns, or unusual symptoms, please return to the clinic promptly to meet with a study nurse or to have a clinical exam.

Final visit:

- At the end of the three months, you will be asked to make have a final clinic visit. At this visit, the study nurse will take 4 swabs from your vagina and cervix to check for infections, and test to measure the amount of HIV in your vagina (as described above)

STI testing:

The study nurse will give you medicine if your test results show that you have a curable reproductive tract infection that can be cured (You must be treated for this infection before you can be enrolled in the study).If you agree, your partner will also be told about the infection and given treatment at no charge. In most cases, you will get your test results and any treatment at your next clinic visit, or you might be called back before your next clinic visit.

During the study, if you have genital ulcer disease, you will be treated for curable infections (syphilis and chancroid) right away, and a test will be conducted after the study to identify which infection caused your ulcer.

You had a pap smear as part of the study screening process, and no cancer was detected. You will have tests for a virus that may cause abnormal pap smears and may lead to cervical cancer. You will not be given these results. It is important that you have regular annual pap smears which is the best way to check for cervical cancer.

Blood testing

Once a month, you will have a blood test for the amount of virus in your blood. For this test about two teaspoons of blood will be taken from your arm. You will have blood drawn three times in this study.

HIV infection

You will be given advice on staying healthy and about how to avoid giving HIV infection to others. You can tell your husband or sex partner about your infection, or the study staff will tell him for you. If you said you would not be sexually active with your husband or steady partner during the study and you plan to become sexually active, it is important that you come to the clinic to talk with the study staff about this right away.

You will be told about medical and social services for people with HIV infection in the area.

You have been referred to Chiang Rai Hospital for medical care. This visit and other medical expenses related to your HIV infection up to 1000 baht will be paid for by the study. If you are part of the government health program in which you pay 30 baht for every medical visit which covers the visit and any care they receive, this money will be given directly to the hospital by a study nurse at the time of your visit by a study nurse for the care you receive; in some cases (like if you pay for your healthcare in another way), the money will be given to you by the study staff if you bring your receipts for medical care you paid for to the clinic. Expenses for HIV related treatment beyond the 1000 baht will have to be paid for by you. Medicines (and the cost for any of these medicines) for treating HIV infection will not be provided to you or your partner as a part of this study.

Interview/Counseling

At each clinic visit:

-You will be asked questions about your sexual activity, gel use and condom use.

-You will get counseling on family planning, safer sex and about HIV infection.

-You will be asked (once a month) what you think about using the gel.

Benefits

There is no cost to you to take part in this study. Benefits that you can gain from being in the study include the limited medical check-up and care already described in the STI testing and HIV testing sections above.

-STI treatment for your partner if needed

-Safer-sex advice at each visit.

-The knowledge that you can help in the early tests of a microbicide gel that some day might help prevent HIV and other STIs. In this way, you are helping other women in your area and in the rest of the world.

Risks and Burdens

You might have burning, itching, pain, or soreness using either study gel. Based on what is known about both gels, however, this is not very likely. Using the microbicide gel could increase your risk of getting certain STIs. This is also not very likely. Other possible risks are:

-Discomfort and/or some bleeding during or after the vaginal exam.

-Soreness or bruising on your arm where blood was drawn.

Possible burdens that you may have while you are in the study include:

-The burden of coming to the clinic each month.

-The stress you may feel if test results show that you have an STI.

-The problems you may face with your partner from using the study products or if you need treatment for an STI.

Reimbursement

At each clinic visit, you will receive 300 baht to cover costs for your time and transportation. . At his clinic visit, your husband/partner will be given 50 baht) for his transportation costs for participating.

Physical Harm

If you are hurt as a result of being in this study, immediate, short-term treatment for STIs and other infections which may be a result of participating in the study will be provided by the investigators. We (CDC, The Population Council, and the Thailand Ministry of Health) do not normally pay for long-term care for harm done to you as a result of being in a research study. Thus, you (or your insurance program) will have to pay for any care that is needed. However, by signing this consent form and agreeing to be in this study, you are not giving up any of your rights. If you believe that you have been harmed, please contact Supaporn Chaikummao at 01-850-4343 or 053-711-827 for information on your rights and advice on how to proceed.

For any conditions which may arise while you are in this safety study, you will be referred to the proper public health services in your area. Except for the HIV testing and STI treatment, no payment or medical care will be given for any member of your family during or after the study.

Whom to Contact in Case of an Emergency

If a medical emergency comes from using the gel or you feel a medical emergency will stop you from taking part in this study, you are to contact Dr. Somsak Supawitkul, Deputy, Office of Preventive Health, Chiang Rai Public Health Office (tel. 711-911, 711-403). Or you can contact Dr. Paisit Witwatwongwana, Staff Obstetrician/Gynecologist, Chiang Rai Hospital (tel. 711-300 ext 213).

Other ways to get testing and treatment if you do not want to be in this study

You can get some, but not all, of the advice, testing, and treatment like that being offered in this study at Chiang Rai Hospital and at other clinics in this area, even if you are not part of this study.

Confidentiality

Your answers to the interview questions, your exam results, and your test results will not be shared with others, such as your husband/regular partner or your doctor, without you saying it’s OK, unless required by law. Most study documents will only have a code number and will not have your name. All study documents will be kept in locked files. We will send you letters to remind you of your follow-up visits. If you miss a visit, or in some cases if you have a positive test result, we will try to contact you by sending letters, telephoning, and/or visiting you or, with your OK, a contact person named by you. We will not put the reason for the visit in the reminder letters or tell it to others.

In order to make sure the study is carried out right, we may ask you to have an interview with a representative of the Population Council during the study so we can understand whether the study is being done well. If someone asks you to talk about the study, you can say “yes” or “no.” If you say “yes,” all facts from the meeting will be kept private.”

This person would check that you know the purpose of the study, the steps in the study, and all possible risks and burdens or benefits from being in the study.

People from the Thai FDA or the US FDA (the groups in charge of medicine use in Thailand and the United States) may ask to see your records. These records will not contain your name.

Being in this study is voluntary

Taking part in this study is up to you. You may stop taking part at any time. You may refuse to answer any of the questions. Your medical care will not be affected if you decide not to take part in the study or if you decide to stop taking part in the study at a later date. If you want to stop taking part in the study, we will ask you if you want to have any more interviews or exams, and about returning the study gel and applicators. The study staff may ask you to stop using the study gel if they think it is better for your health, or if they feel you cannot follow the steps of this study. In this case you will be asked to continue to come to the clinic for interviews, exams, and testing. If we learn anything new about the study gels during the study that you might want to know, the study staff will tell you.

The Population Council may stop the trial at any time if they feel it is necessary.

If you have questions about the study or if you believe you have been injured as a result of being in the study

You can call Khun Supaporn Chaikummao at the Chiang Rai Health Club (tel. 711-234) or (tel: 01-8504343 cell, 24 hours). Or you can call Dr. Somsak Supawitkul, Deputy, Office of Preventive Health, Chiang Rai Public Health Office (tel. 711-911, 711-403).

If you have questions about whether you should agree to be in the study or about the way the study is being done

You can talk to Wanida Maneerut, Nurse, Chiang Rai Provincial Public Health Office Tel 711-911 ext. 110.

If you have questions about your rights as a study subject

You can call the Secretary to Ethical Review Committee, at the Ministry of Public Health in Nonthaburi (tel. 02-591-8249; fax. 02-591- 8245).

Participant’s statement

-I have had a chance to ask questions about the study. My questions have been answered.

-I have had a chance to ask questions and my questions have been answered to my satisfaction about the purpose of the study, the study steps, and the risks and benefits.

-I agree to take part in this study, to follow the study steps, and to come to all study visits as best as I am able.

-I have had a chance to ask questions and my questions have been answered to my satisfaction about the fact that I may stop taking part in this study at any time without affecting my right to receive regular medical care.

□ Yes, I would like to be contacted after the end of the study with the results of any genital ulcer disease testing.

□ No, I do not want to be contacted after the end of the study with the results of genital ulcer disease testing.

Name of Participant: ________________________________

_______________ _________________________________ ____________

Date Signature of Participant * (thumbprint)

*In case the woman is not able to sign this form, this attests that the consent form has been read and explained by a member of the research staff, and that the woman has fixed her thumbprint as consent.

We would like to keep any blood or other fluids that are left over after we do your tests. We may use these samples for future tests such as other infections; however, we will not do any human genetic testing on your specimen. We will keep these samples with some facts about you such as your age and sex. We will not put your name on the samples so we would not be able to report the results of any future tests back to you.

□ Yes, I have had a chance to ask questions and my questions have been answered to my satisfaction about the fact that any blood or other fluids that are left over after my tests are done will be kept.

□ No, I do not want any blood or other fluids that are left over after my tests are done to be kept.

Name of Participant: ________________________________

_______________ _________________________________ ____________

Date Signature of Participant * (thumbprint)

*In case the woman is not able to sign this form, this attests that the consent form has been read and explained accurately by a member of the research staff, and that the woman has fixed her thumbprint as consent.

Study Team Member’s Statement

I, the undersigned, have explained to the volunteer in a language that she understands, the procedures to be followed in the screening and the study, the risks and benefits involved, and the obligations of the study team.

_______________ _________________________________

Date Signature of Study Team Member

______________ _________________________________

Date Signature of Witness to the Above

Signatures and Explanations

**Appendix 4: Table 1. Study visits, based upon ~28 day menstrual cycle**

| 1 2 3 4 Menstrual bleeding | 5 | 6 | 7 | 8 | 9 | 10 | 11 | 12 | 13 | 14 | 15 | 16 | 17 | 18 | 19 | 20 | 21 | 22 | 23 | 24 | 25 | 26 | 27 | 28 |
| --- | --- | --- | --- | --- | --- | --- | --- | --- | --- | --- | --- | --- | --- | --- | --- | --- | --- | --- | --- | --- | --- | --- | --- | --- |
| Washout period | | | 7 day product use | | | | | | | Washout period | | | | | | | | | | | | | | |

↑ ↑ ↑

Clinic visits Enrollment(First Baseline) Follow-up #1(Day 7) Follow-up #2 (Day 14)

| 1 2 3 4 Menstrual bleeding | 5 | 6 | 7 | 8 | 9 | 10 | 11 | 12 | 13 | 14 | 15 | 16 | 17 | 18 | 19 | 20 | 21 | 22 | 23 | 24 | 25 | 26 | 27 | 28 |
| --- | --- | --- | --- | --- | --- | --- | --- | --- | --- | --- | --- | --- | --- | --- | --- | --- | --- | --- | --- | --- | --- | --- | --- | --- |
| Washout period | | | 7 day product use | | | | | | | Washout period | | | | | | | | | | | | | | |

↑ ↑ ↑

Clinic visits Baseline (Day 0/28) Follow-up #1(Day 7) Follow-up #2 (Day 14)

| 1 2 3 4 Menstrual bleeding | 5 | 6 | 7 | 8 | 9 | 10 | 11 | 12 | 13 | 14 | 15 | 16 | 17 | 18 | 19 | 20 | 21 | 22 | 23 | 24 | 25 | 26 | 27 | 28 |
| --- | --- | --- | --- | --- | --- | --- | --- | --- | --- | --- | --- | --- | --- | --- | --- | --- | --- | --- | --- | --- | --- | --- | --- | --- |
| Washout period | | | 7 day product use | | | | | | | Washout period | | | | | | | | | | | | | | |

↑ ↑ ↑

Clinic visits Baseline (Day 0/28) Follow-up #1 (Day 7) Follow-up #2 (Day 14)

| 1 2 3 4 Menstrual bleeding | 5 | 6 | 7 |
| --- | --- | --- | --- |
| Washout period | | | |

↑

Clinic visits Day 28, 3^rd^ arm

**Appendix 5: Table 2. Schedule of clinic visits and data collection**

| Study visit | Screen #1 | CRH referral visit | Screen #2 | Male  partner  visit | Enrollment | Follow-up #1 | Follow-up #2 | Baseline | Closing |
| --- | --- | --- | --- | --- | --- | --- | --- | --- | --- |
| Study day | ~ (-30) | ~ (-21) | ~ (-14) |  | Day 0 | Day 7 | Day 14 | Day 0/28 | Day 28,  3^rd^ arm |
| Menstrual day (~28 day) |  |  | Mid-cycle |  | 3-5 days after menses | 10-12 | 17-19 | 3-5 days after menses |  |
| *Specimen collection* |  |  |  |  |  |  |  |  |  |
| HIV test | X |  |  | X |  |  |  |  |  |
| CD4 count | X |  |  |  |  |  |  |  |  |
| RPR | X |  |  |  |  |  |  |  |  |
| Urine preg test |  |  | X |  | X |  |  | X |  |
| HIV viral load |  |  |  |  | X |  |  |  |  |
|  |  |  |  |  |  |  |  |  |  |
| Pap smear |  |  | X |  |  |  |  |  |  |
| Gram stain, ph |  |  | X |  | X | X | X | X | X |
| Gonorrhea, chlamydia |  |  | X |  |  |  |  |  | X |
| Trich In-pouch test |  |  | X |  |  |  |  |  | X |
| Pelvic exam |  |  | X |  | X | X | X | X | X |
| HPV |  |  | X |  |  |  | X |  |  |
|  |  |  |  |  |  |  |  |  |  |
| Colpo |  |  |  |  | X (baseline)  X (15 min) | X |  | X (baseline)  X (15 min) | x |
| CV swab for HIV |  |  |  |  | X (baseline)  X (15 min.) | X | X | X (baseline)  X (15 min.) | X |
|  |  |  |  |  |  |  |  |  |  |
| CVL |  |  |  |  | X (baseline)  X (15 min.) | X | X | X (baseline)  X (15 min.) | X |
| Product application |  |  |  |  | X |  |  | X |  |

**Appendix 6: Table 3. Forms schedule**

| Study visit | Screen #1 | CRH referral visit | Screen #2 | Male  partner  visit | Enrollment | Follow-up #1 | Follow-up #2 | Baseline | Closing visit |
| --- | --- | --- | --- | --- | --- | --- | --- | --- | --- |
| Study day | ~ (-30) | ~ (-21) | ~ (-14) | Prior to enrollment | Day 0 | 7 | 14 | 0/28 | 28 of last arm |
| *Informed consent forms* |  |  |  |  | 3-5 days after menses |  |  |  |  |
| Screening Informed consent | X |  |  |  |  |  |  |  |  |
| Male informed consent |  |  | X |  |  |  |  |  |  |
| Enrollment Informed consent |  |  |  |  | X |  |  |  |  |
| *Study visit forms* |  |  |  |  |  |  |  |  |  |
| Eligibility checklist | X |  |  |  |  |  |  |  |  |
| Clinician screening form |  |  | X |  |  |  |  |  |  |
| CRH referral letter |  | X |  |  |  |  |  |  |  |
| CRH Assessment form |  | X |  |  |  |  |  |  |  |
| Male HIV test form |  |  | X |  |  |  |  |  |  |
| Comprehension test | X |  |  |  |  |  |  |  |  |
| Enrollment form |  |  |  |  | X |  |  |  |  |
| Enrollment interview |  |  |  |  | X |  |  |  |  |
| Clinician study form |  |  |  |  | X | X | X | X | X |
| Acceptability questionnaire |  |  |  |  |  | X* |  |  |  |
| Interview follow-up form |  |  |  |  |  | X | X | X | X |
| Closing form |  |  |  |  |  |  |  |  | X |
| *Additional forms* |  |  |  |  |  |  |  |  |  |
| Master checklist |  |  |  |  | X | X | X | X | X |
| Product distribution log |  |  |  |  | X |  |  | X |  |
| Medication log |  |  |  |  | X | X | X | X | X |
| Applicator usage log |  |  |  |  |  |  |  |  |  |
| Study gel interruption form |  |  |  |  |  |  |  |  |  |
| Adverse events form |  |  |  |  |  |  |  |  |  |
| Severe adverse events form |  |  |  |  |  |  |  |  |  |

*Only on Day 7 at end of second gel month

**Appendix 7: Table 4. Laboratory tests, specimens, and processing laboratories**

| *Purpose* | *Test* | *Specimen* | *Volume* | *Lab processing* |
| --- | --- | --- | --- | --- |
| HIV | EIA and W. Blot | Blood (serum) | 10ml (also for syphilis serology) | TUC |
| Syphilis | RPR (TPPA if RPR is reactive) | Blood (serum) | “ | TUC |
| CD4 count | FACScan + SimulTest | Blood (EDTA plasma) | 5 - 7mL (also for RNA viral load) | TUC |
| HIV viral load | Roche Amplicor HIV-RNA | Blood (EDTA plasma) | (See above) | TUC |
| *N. gonorrhoeae* | COBAS Amplicor CT/NG PCR | Endocervical ( ½ swab ) |  | TUC |
| *C. trachomatis* | COBAS Amplicor CT/NG PCR | Endocervical ( ½ swab ) |  | TUC |
| *T. vaginalis* | InPouch wet mount and culture | Vaginal swab (1) |  | Chiang Rai |
| *B. vaginalis* | gram stain (Nugent criteria) | Vaginal swabs (1) |  | Chiang Rai |
| PH | pH strip | Vaginal swab (1) |  | Chiang Rai |
| *C. albicans* | Gram stain | Vaginal swab (0) |  | Chiang Rai |
| *HSV, H. ducreyii,*  *T. pallidum* | Multiplex GUD PCR | Ulcer swab (1) |  | CDC |
| Cervical atypia | Pap smear | Cervical swab (1) |  | Chiang Rai |
| HPV | PCR | Cervical swab (1) |  | TUC  CDC |
| Cervico-vaginal HIV:  - RNA Q PCR (VL*)    - Spliced HIV-1mRNA  -infectious virus | Amplicor v.1.5  Amplicor 1.5  MAGI assay | CVL supernant  Vaginal swab (2)  CVL cell pellets  CVL supernant | 1 mL  2 swabs  whole pellet  1-2ml | TUC  TUC  CDC  CDC |
| **Semen detection** | Abacus Diagnostics | Vaginal swabs (1) | 200 mcl | Chiang Rai |
| Pregnancy | HCG |  | 5-10ml | Chiang Rai |

*VL ‘ Viral Load
